# A Path Analysis Model of Protection and Risk Factors for University Academic Stress: Analysis and Psychoeducational Implications for the COVID-19 Emergency

**DOI:** 10.3389/fpsyg.2021.562372

**Published:** 2021-08-13

**Authors:** Jesús de la Fuente

**Affiliations:** ^1^School of Education and Psychology, University of Navarra, Pamplona, Spain; ^2^School of Psychology, University of Almería, Almería, Spain

**Keywords:** academic stress, protection and risk factors, 3P model, SLPS competency model, SRL vs. ERL theory, university students, COVID-19

## Abstract

The aim of this research was to empirically validate hypothesized predictive relationships of protection and risk factors for experiencing academic stress. A synthesis of models—the presage–process–product model; the studying, learning and performing under stress competency model; and self- vs. external-regulatory theory—underlies the investigation and is important for assessment and guidance in stress situations within the university context. Over the course of an academic year, a sample of 564 Spanish university students voluntarily completed validated questionnaires, in an online format, on several psychological variables connected to academic stress. Correlational analysis and the path analysis model, within an *ex post facto* design, were used to build empirical models of the presage–process–product factors that constitute protection or risk factors in academic stress. Two statistically acceptable models appeared: one with protection factors and another with risk factors in predicting and preventing academic stress at a university. These results support the need for psychology units at university that have a preventive, health and education focus, going beyond the merely clinical. Focus on an individual is insufficient, given that there are also contextual factors that predispose academic stress. Discussion, conclusions, and implications for assessment and intervention in academic stress in university students and teachers, within the present COVID-19 crisis, are offered.

## Introduction

Human beings require learning experiences in order to restructure their knowledge and their ways of interacting with reality; today, COVID-19 has become such an experience—unusual, unexpected, but common among us all. In the field of healthcare, it is an object of analysis and learning. It is obvious that COVID-19 has all the components of a health and medical-biological emergency, just as what was declared by the WHO. The configuration, functionality, and structure of this fast-spreading biological entity are not yet clearly understood. Knowledge has been lacking about its primary care, through conventional pharmacological prevention (vaccines), and its secondary or tertiary care (pharmacological treatment, ventilators, etc.). As a consequence, the disease is now pandemic and growing by geometric progression.

To evaluate and intervene in behavioral variables (psychoeducational and psychosocial) is the business of psychology as a behavioral science. It is time to recognize that many health-related issues have both a medical-biological component and a psychosocial component (behavioral, personal, and contextual). We must learn that medicine, biology, and psychology should work together on epidemiological and health-related issues from an integrated, biopsychosocial model (de la Fuente, 2020a; Frazier, 2020).

In the context of today, educational psychology—as a specialized branch of scientific knowledge in psychology—can contribute its own elements and models in the realm of academic stress. This article thus has a 3-fold aim: (1) present a conceptual–synthesis model or heuristic based on previous conceptual models that have provided evidence (Slavin, 2019); (2) empirically demonstrate the hypothesized relationships in university students; (3) present implications and proposals for intervention to help educational psychologists evaluate and advise students, teachers, and university institutions during the COVID-19 health crisis. An example of this purpose is the *Research Topic* in which the present research report is included (de la Fuente et al., 2021a).

In a complementary fashion, this exploratory study seeks to offer an empirical and conceptual synthesis of different theoretical models in the line of research that analyzes variables connected with stress behavior in the university context. A few partial contributions have been put forward to date, which we seek to integrate into this final model or heuristic.

### The 3P Model for Analysis Within the COVID-19 Health Emergency

The 3P (presage–process–product) model, or theory of Biggs of student approaches to learning (SAL) (Biggs, 1970a, 1985, 1993, 1999), is an essential conceptual heuristic for addressing the prevention of academic stress, particularly in the context of the COVID-19 health crisis. It presents a systemic view of university teaching and learning processes and has become one of the seminal models that are most prevalent in the literature (Barattucci et al., 2017; Ginns et al., 2018; Kember et al., 2020):

1) *Presage variables*. Evidence has demonstrated the existence of different *presage* variables of university learning and academic achievement. Biggs himself proposed influence coming from personality factors (Biggs, 1970a) and factors of the faculty context (Biggs, 1970b) as precursors to the learning approach of the students at a university (Chamorro-Premuzic et al., 2007; Ginns et al., 2018). Positive psychology has recently contributed new elements for consideration, such as positivity as dispositional optimism (Caprara and Steca, 2005; Caprara et al., 2006, 2009, 2010, 2011; Alessandri et al., 2012). Relationships have also appeared between personality and academic confidence (Sander and de la Fuente, 2020).
2) *Process variables*. The core research, using this model, has historically focused on learning approaches as an essential process variable (Biggs, 1972, 1973, 1976, 1978, 1985, 1987). Classical cognitive research established associations and predictive relationships with cognitive variables like learning strategies, metacognitive processes, and self-regulated learning (Heikkilä and Lonka, 2006; de la Fuente et al., 2008). This paradigm has evolved toward the study of emotional and affective factors in our day, establishing relationships between learning approaches and several variables of this type (Trigwell and Ashwin, 2003; Trigwell, 2006, 2012; Trigwell et al., 2012, 2013).
3) *Product variables*. Finally, the *product* variable is understood to be achievement or satisfaction, and previous research showed a predictive relationship between learning approaches and achievement (Karagiannopoulou et al., 2018).

The 3P model can serve as a general heuristic for evaluating complex realities, such as that of COVID-19 in the university context. However, despite the wide range of evidence and research on this topic, an essential aspect of reality has not been thoroughly analyzed, namely, the high level of environmental and personal stress that exists in this context. This limitation has given rise to other complementary heuristics that address elements of the model that can be improved. Such is the case of the *SLPS Competency Model* (de la Fuente, 2015a).

### Competence in Studying, Learning, and Performing Under Stress as a Model for Analysis Within the COVID-19 Health Emergency

#### The (Original) SLPS Competency Model (V.1)

The educational psychology model of competence in *studying, learning, and performing under stress* (SLPS) (de la Fuente, 2015a) is based conceptually on the Gagné instructional model (Gagné, 1985), taking into account three levels of learning that are required to be competent. It focuses on the process variables of the 3P model (Biggs, 1993), since it establishes behaviors that make up appropriate repertories for dealing with academic stress situations. Given the current COVID-19 crisis, it seems reasonable that this model can be useful to evaluate and intervene with university students who so require. See Table 1.

**Table 1:** The competency model of studying, learning, and performing under stress, SLPS original (de la Fuente, 2015a).

**Knowing (knowledge):** • Facts • Concepts • Principles **Knowing how (skills):** • Instrumental skills: written and oral skills • Learning and study skills: study skills and techniques • Meta-cognitive skills for study: **learning approaches** • Meta-emotional skills for managing stress: **coping strategies** • Meta-behavioral skills for managing stress: **self-regulation vs. procrastination strategies** • Meta-motivational skills for managing stress: **resilience Knowing how to be (attitudes):** • Achievement emotions: **positives vs. negatives** • Attitudes and values: **academic behavioral confidence** •Emotional motivation: **engagement- burnout**

*The variables on which this research has focused are highlighted in bold*.

The *learning approaches* variable was considered a meta-learning variable by Biggs himself (1985). Both the theory of learning approaches (Biggs, 1993; Asikainen and Gijbels, 2017) and its assessment instrument (Biggs et al., 2001) have become established internationally. Recent research has consistently shown that *deep approach* is associated with better learning and achievement at a university, while *surface approach* is associated with poorer university learning and achievement (Cetin, 2015; Asikainen and Gijbels, 2017). Learning approach has recently been related to coping strategies and resilience, with deep approach related to problem-focused strategies and high resilience, and surface approach to emotion-focused strategies and low resilience (de la Fuente et al., 2017a; Banerjee et al., 2019). Relationships have recently been established between *learning approach* (deep vs. surface) and *achievement emotions* (positive vs. negative), respectively (de la Fuente et al., 2020e).

*Self-regulation*, as a meta-behavioral variable, has also been related to a number of variables: in positive association with type of coping strategies used (de la Fuente, 2020a,b), positive achievement emotions (de la Fuente et al., 2020c), academic behavioral confidence (de la Fuente et al., 2020f), and deep learning approach (de la Fuente et al., 2021c), and in negative association with procrastination (Garzón-Umerenkova et al., 2018).

*Coping strategies*, as a meta-affective variable, have also shown a relation to the states of engagement burnout (de la Fuente et al., 2015a). Moreover, a recently proposed relationship model, including achievement emotions, emotion- vs. problem-focused coping strategies, and ultimate state of engagement burnout, has also acted as a potential 2-fold mechanism in positive vs. negative perfectionism (de la Fuente et al., 2020b). Coping strategies have also been related to the self-regulation characteristics of students (Amate-Romera and de la Fuente, 2021).

Resilience also has been studied as a meta-motivational variable, mediating between personality characteristics and perceived stress (de la Fuente et al., 2021f). Other studies examine its predictive value for coping strategies and the motivational states of engagement burnout (de la Fuente et al., 2021d).

Achievement emotions likewise have been studied widely in recent research, with much important evidence. Positive and negative relationships have been verified in different stress situations according to the source of the stress triggers (related to class, study time, or testing) (de la Fuente et al., 2020c).

Regarding *academic behavioral confidence* (Sander and Sanders, 2006, 2009; Sander, 2009; Sander et al., 2011), prior research showed its positive relationship to deep learning approach and to academic achievement (de la Fuente et al., 2013). More recently, a relationship to positive achievement emotions has also been found (Sander and de la Fuente, 2020).

#### The (Adapted and Integrated) SLPS Competency Model as a Buffering Variable When Facing Academic Stress (V.2)

The *competency model for studying, learning, and performing under stress*, SLPS (the adapted and integrated model; de la Fuente, 2021) assumes that, if a university student has an adequate level of the learning behaviors that make up this competency, these behaviors will act as *protective factors* or *buffers* against stress. The student will be able to adequately cope with academic stress situations and ultimately have fewer learning problems and stress symptoms (de la Fuente, 2015b,c). However, there are also risk factors that can predispose a greater experience of academic stress.

**Figure 1:**
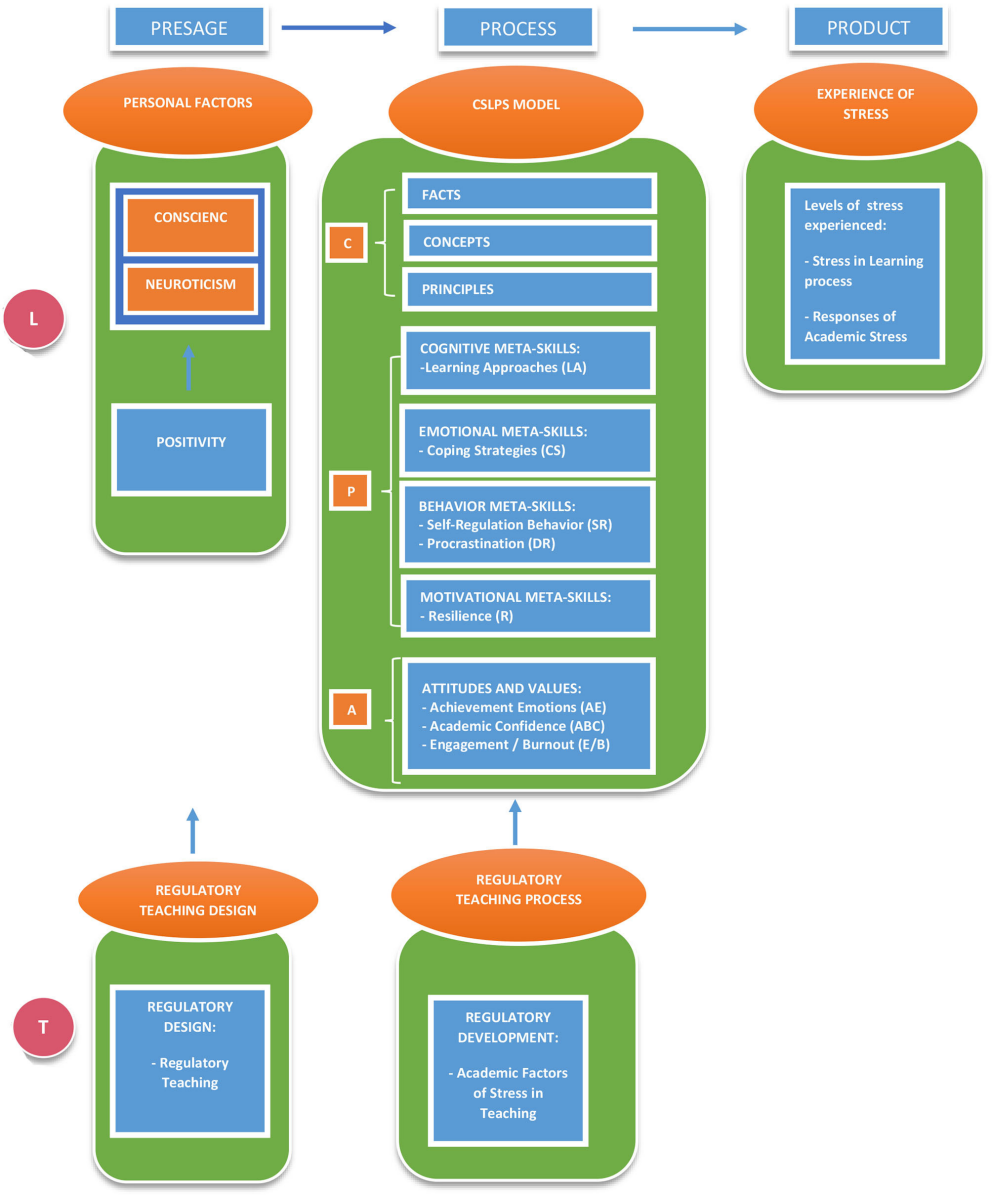
Competency model for studying, learning, and performing under stress, SLPS: an adapted and integrated model, v.2. (de la Fuente, 2021), with variables of this study. L, learning process variables; T, teaching process variables; C, conceptual factors; P, procedural factors; A, attitudinal factors.

Despite the goodness of this model, it still underplays *contextual factors* (the design and development of teaching) that can also carry weight as protection or risk factors in experiencing stress. For this reason, contributions from SRL vs. ERL theory have also been taken into account (de la Fuente, 2017). Figure 1 shows a graphic representation of this adapted model in the context of the former models.

### SRL vs. ERL Theory in the COVID-19 Health Emergency

#### The SRL vs. ERL Model as a Heuristic for Analyzing Stress Factors in the University Teaching and Learning Process

The *theoretical model* entitled *SRL vs. ERL theory* (de la Fuente, 2017; de la Fuente et al., 2019a) seeks to straightforwardly identify the possible combinations of internal and external regulations that can occur in any university teaching–learning process. Basically, *students* are assumed to have different levels of behavioral self-regulation (high, medium, and low); they take on a given learning process from different starting points (with regulating, non-regulating, or dysregulating behaviors). In the same degree, teachers can show diverse teaching behavior in regard to external regulation (high, medium, and low); their teaching behaviors affect learning in ways that can be regulatory, non-regulatory, or dysregulatory (Pachón-Basallo et al., 2021). These two typologies in students and teachers are then combined in the teaching-learning process, giving rise to multiple interactions. Recent research has presented a heuristic that organizes the different possible interactions and has also tested their effects, with consistent evidence in relation to learning approaches and academic achievement (de la Fuente et al., 2020d), and to the factors and symptoms of stress (de la Fuente et al., 2020f).

Prior evidence reported on (1) the effect of the levels of self-regulation of students (high, medium, and low) on their learning behaviors and on emotional resources that they engage in during university learning; (2) the effect of the level of regulatory teaching (high, medium, and low) on learning behaviors and on the emotional resources that students engage in during university learning; (3) the combined effect of the different possible interactions between student and teacher regulatory levels, representing these as a consistent, increasing or decreasing linear function, according to the variable analyzed. Thus, the combination of different levels of regulation—from the lowest and most dysregulatory to the highest and most regulatory—has been found to determine the positive and negative achievement emotions of university students, as well as emotion- or problem-focused coping strategies (de la Fuente et al., 2019b, 2020a,b). This combination, moreover, has been found to determine learning approaches, perceived satisfaction, and personal achievement, as well as academic behavioral confidence and procrastination (de la Fuente et al., 2020b,c). In turn, characteristics of the teaching process determine the factors and symptoms of the stress of the students when learning (de la Fuente et al., 2021a,c,e,f). In summary, the stress reactions of university students depend on *personal* factors and also on *contextual* factors or the type of teaching process deployed.

This theoretical model proposes *effective* or *regulatory teaching* as a *buffering* factor against academic stress. Insofar as the teaching process is regulatory or effective, it will minimize the effect of stress during the learning process, particularly in this exceptional context of COVID-19. The teaching process during this period should be regular, clear, and predictable. Any radical, disorienting changes or adjustments during this period will not contribute to a buffering effect against stress but will become stress triggers (Barattucci, 2017; de la Fuente et al., 2017b). This schema has also been applied to psychoeducational behavior analysis in the COVID-19 health emergency (de la Fuente et al., 2021b).

**Figure 2:**
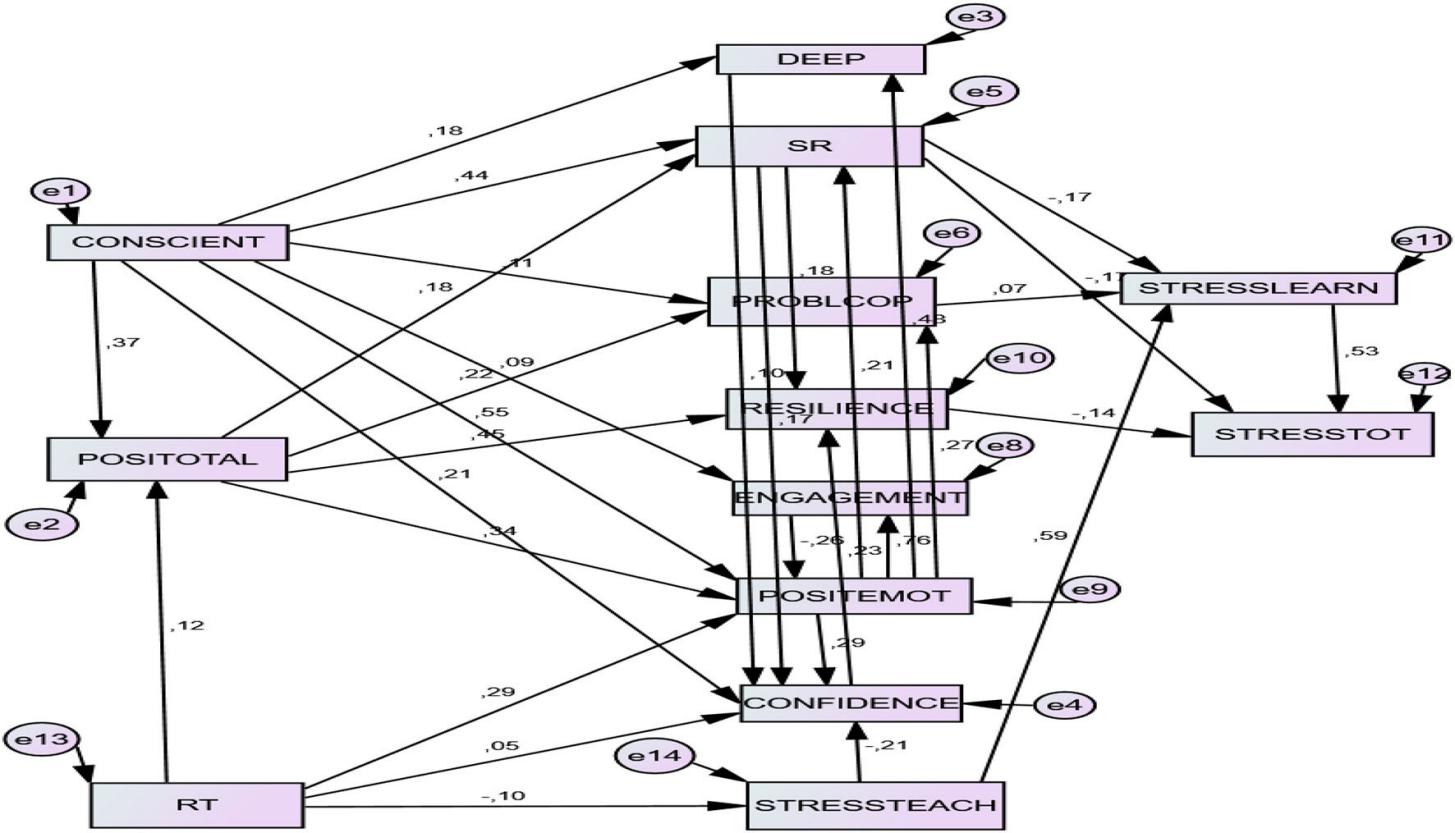
A structural predictive model of protective factors against academic stress (Model 2). CONSCIENT, conscientiousness; POSITOTAL, Positivity; DEEP, deep approach; CONFIDENCE, academic behavioral confidence; SR, self-regulation; PROBLCOP, problem-focused coping; POSITEMOT, positive achievement emotions; RT, regulatory teaching; STRESSTEACH, stress factors of teaching process; STRESSLEARN, stress factors of learning process; STRESSTOT, symptoms of stress.

The three preceding models have been conceptually merged in an updated, integrated version (de la Fuente, 2021; V.2), taking into account variables from the teaching process, which were not present in the original model (de la Fuente, 2015a). See Figure 2.

### Aims and Hypotheses

Based on the conceptual synthesis presented, the *aim* of this research was to empirically analyze the hypothesized relationship between personal characteristics of students (*presage* variables); their *competency for learning under stress*, as a protective or buffering variable against stress (a *process* variable); and their learning difficulties and final stress levels (a *product* variable) in order to establish implications for evaluation and intervention in a situation with high academic stress, such as the COVID-19 health emergency. Based on previous research and the existing evidence, the following relationships were hypothesized in situations of academic stress:

*Hypothesis 1*. There will be a *structural predictive relationship, protecting* against academic stress, that comprises the following: (1) *presage factors*, including the personality component of *conscientiousness* and its associated positivity; (2) *process factors* with a buffering effect, that are part of the competence for coping with stress (meta-cognitive, meta-emotional, meta-motivational and meta-behavioral, and attitudinal variables); (3) *product factors*: a low level of learning-related or academic stress as final dependent variables of the prediction.
*Hypothesis 2*. The relationship established in hypothesis 1 will be modulated positively by factors of the teaching context: (1) *Presage factors*: high regulatory teaching; (2) *process factors:* low stress factors from difficulties in the teaching process: (3) *product factors*: a low level of learning-related or academic stress as final dependent variables of the prediction.
*Hypothesis 3*. There will be a *structural predictive relationship* of *vulnerability* to academic stress that comprises: (1) *presage factors*, including the personality component of *neuroticism* and its associated lack of positivity; (2) *process factors* that pertain to a lack of competence for coping with academic stress (lack of meta-cognitive, meta-emotional, meta-motivational and meta-behavioral, and attitudinal variables); (3) *product factors*: a high level of learning-related or academic stress as final dependent variables of the prediction.
*Hypothesis 4*. The relationship established in Hypotheses 3 will be modulated negatively by factors of the teaching context: (1) *Presage factors*: low regulatory teaching; (2) *process factors:* high stress factors from difficulties in the teaching process: (3) *product factors*: a high level of learning-related or academic stress as final dependent variables of the prediction.

## Method

### Participants

The participants were 564 students enrolled in Psychology and Primary Education degrees at two Spanish universities. Their ages ranged from 19 to 25, with a mean age of 22.35 (^σ^*X* = 7.1) years. Students age 26 and older were excluded. About 80.3% were women and 19.7% were men. Sampling was incidental and not probabilistic. The students from 20 different academic subjects completed the inventories. An incidental, non-randomized study design was used. Each Guidance Department of the universities invited participation from teachers, and the teachers invited their students to participate on an anonymous, voluntary basis. Each course (subject) was considered one specific teaching–learning process. The students completed the questionnaires online for one subject over one academic year. Only the students who voluntarily wished to participate did so.

### Instruments

#### Presage Factors

*Conscientiousness and neuroticism* (*personal factors*) were assessed, using the *big five questionnaire* BFQ-N (del Barrio et al., 2006) based on Barbaranelli et al. (2003), and adapted for young university students (de la Fuente, 2014a). Confirmatory factor analysis (CFA) reproduced a five-factor structure corresponding to the big five model. The results showed adequate psychometric properties and acceptable fit indices. The second-order confirmatory model showed a good fit [chi-square = 38.273; degrees of freedom (20–15) = 5; *p* < 0.001; NFI = 0.939; RFI = 0.917; IFI = 0.947; TLI = 0.937, CFI = 0.946; RMSEA = 065; HOELTER = 2,453 (*p* < *0.0*5) and 617 (*p* < *0.0*1)]. The total scale also showed good internal consistency [alpha = 0.956; Part 1 = 0.932, Part 2 = 0.832; Spearman–Brown = 0.962; Guttman = 0.932].

*Positivity* (*personal factor*). Measured by the *Escala de Positividad* (positivity scale) (Caprara et al., 2012). This scale contains 10 items on a five-point Likert response scale. The Spanish validation data for our sample produced acceptable values [chi-square = 308.992; degrees of freedom (44–20) = 20; *p* < *0.0*01; NFI = 0.901; RFI = 0.894; IFI = 0.912 TLI = 0.923, CFI = 0.916; RMSEA = 0.085; HOELTER = 260 (*p* < 0.05) and 291 (*p* < 0.01)]. The total scale also showed good internal consistency (alpha = 0.893; Part 1 = 0.832, Part 2 = 0.813; Spearman–Brown = 0.862; Guttman = 0.832).

#### Process Factors

##### Learning Variables

*Learning approaches* (a meta-cognitive factor) were measured by the revised two-factor *study process questionnaire, R-SPQ-2F* (Biggs et al., 2001). Twenty items measure two dimensions: deep learning approach (e.g., “I find that, at times, studying gives me a feeling of deep personal satisfaction”) and surface learning approach (e.g., “My aim is to pass the course while doing as little work as possible”). Students answer the items on a five-point Likert scale from 1 (“rarely true of me”) to 5 (“always true of me”). The R-SPQ-2F was translated into Spanish, adapted for cultural differences, independently back-translated, and further modified where needed. Using a Spanish sample, Justicia et al. (2008) showed a confirmatory factor structure similar to that of Biggs et al. (2001)—a first-order structure of two factors. These authors also reported acceptable reliability coefficients. In this study, confirmatory factor analysis produced a second factor structure with two factors (chi-square = 2,645.77; df = 169, CFI = 0.95, GFI = 0.91, AGFI = 0.92, RMSEA = 0.07). In the present study, Cronbach alpha reliability coefficients were acceptable (Deep α = 0.81; Surface α = 0.77) and similar to what the original authors found (omega index = 0.85).

*Self-regulation behavior* (a meta-behavior factor). This variable was measured, using the *short self-regulation questionnaire* (SSRQ) (Miller and Brown, 1991). The Spanish adaptation was previously validated in Spanish samples (Pichardo et al., 2014, 2018), showing acceptable validity and reliability, with values similar to the English version. Four factors (goal setting-planning, perseverance, decision-making, and learning from mistakes) were measured by a total of 17 items (all with saturations >0.40). The confirmatory factor structure is consistent (chi-square = 250.83, df = 112, CFI = 0.90, GFI = 0.92, AGFI = 0.90, RMSEA =0.05). Internal consistency was acceptable for the questionnaire total (α = 0.86) and for three factors: goal setting-planning (α = 0.79), decision-making (α = 0.72), and learning from mistakes (α = 0.72). The perseverance factor showed low internal consistency (α = 0.63); omega index = 0.75.

*Procrastination* (a negative meta-behavior variable). For this variable, we used a validated Spanish version of the *procrastination assessment scale-students* (PASS) (Garzón-Umerenkova and Gil-Flores, 2017). The original scale by Solomon and Rothblum (1984) consists of 44 items under two sections. The first section uses 18 items to assess procrastination frequency. Our study made use only of the second section, items 19 to 44, which investigates the cognitive-behavioral reasons for procrastination. On the five-point answer scale, 1 means “It does not reflect my motives whatsoever,” 3 means “It reflects my motives to an extent,” and 5 means “It reflects my motives completely.” In its validation for Spain, a language adjustment was made, and adequate reliability values were obtained (Cronbach's alpha from 0.71 to 0.82; omega index = 0.76). The confirmatory model showed a good fit [chi-square = 944,633; degrees of freedom (350–85) = 265; *p* < *0.0*01; NFI = 0.921; RFI = 0.915; IFI = 0.936; TLI = 0.926, CFI = 0.932; RMSEA = 0.032; HOELTER = 533 (*p* < 0.05) and 565 (*p* < 0.01)].

*Coping strategies* (meta-emotional factor). The *coping strategies scale* (*EEC*) was used in its original version (Sandín and Chorot, 2003), as validated for university students (de la Fuente, 2014b). The Lazarus and Folkman questionnaire (1984) and coping assessment studies by Moos and Billings (1982) were foundational to this scale, constructed according to theoretical-rational criteria. The original instrument contained 90 items. The validation produced a first-order structure with 64 items and a second-order structure with 10 factors and two dimensions, both of them were significant. The dimensions showed adequate fit values [Chi-square = 878.750; degrees of freedom (77–34) = 43, *p* < 0.001; NFI = 0.901; RFI = 0.945; IFI = 0.903, TLI = 0.951, CFI = 0.903]. The measures confirming reliability were Cronbach alpha values of 0.93 (complete scale), 0.93 (first half) and 0.90 (second half), Spearman-Brown of 0.84 and Guttman 0.80, Omega index = 0.86. Eleven factors and two dimensions make up the questionnaire: (1) *Dimension: emotion-focused coping*: F1. Fantasy distraction; F6. Help for action; F8. Preparing for the worst; F9. Venting and emotional isolation; F11. Resigned acceptance; and (2) *Dimension: problem-focused coping*: F2. Help seeking and family counsel; F5. Self-instructions; F10. Positive reappraisal and firmness; F12. Communication of feelings and social support; F13. Seeking alternative reinforcement.

*Resilience* (a meta-motivational factor) was measured, using the CD-RISC scale (Connor and Davidson, 2003) in its validated Spanish version (Manzano-García and Ayala-Calvo, 2013). Adequate reliability and validity values were obtained in Spanish samples, and a five-factor structure: F1: Persistence/tenacity and a strong sense of self-efficacy (tenacity); F2: Emotional and cognitive control under pressure (stress); F3: Adaptability/ability to bounce back (change); F4: Perception of control (control), and F5: Spirituality. The confirmatory model showed a good fit [chi-square = 1,619,170; degrees of freedom (350–85) = 265; *p* < *0.0*01; NFI = 0.929; RFI = 0.948; IFI = 0.922; TLI = 0.908, CFI = 0.920; RMSEA = 0.063; HOELTER = 240 (*p* < 0.05) and 254 (*p* < 0.01); Cronbach alpha = 0.88; omega index = 0.85)].

*Learning related emotions* (an attitudinal factor) were measured by the achievement emotions questionnaire (AEQ) (Pekrun et al., 2002; Perry et al., 2005), with scales for nine different emotions (enjoyment, hope, pride, relief, anger, anxiety, hopelessness, shame, and boredom), measured along two axes. The nine different emotions include emotions occurring during activity (enjoyment, boredom, and anger), prospective outcome emotions (hope, anxiety, and hopelessness), and *retrospective* outcome emotions (pride, relief, and shame). The two axes address *valence*, whether positive or negative emotions, and *activation*, where emotions can be either activating or deactivating. The four resulting quadrants are able to classify the emotions as either: (1) *positive activating*: enjoyment, hope, and pride; (2) *positive deactivating*: relief; (3) *negative activating*: anger, anxiety, and shame; or (4) *negative deactivating*: hopelessness and boredom. In this sample, confirmatory factor analysis (CFA) reproduced a structure that corresponds to the AEQ model:

1) *Achievement emotions pertaining to class* (Paoloni, 2015). The results showed adequate psychometric properties and acceptable fit indices. The confirmatory model showed a good fit [chi-square = 843.028; degrees of freedom (44–25) = 19; *p* < *0.0*01; NFI = 0.954; RFI = 0.967; IFI = 0.953; TLI = 0.958, CFI = 0.971; RMSEA = 0.081; HOELTER = 156 (*p* < 0.05) and 158 (*p* < *0.0*1). Internal consistency for the total scale was good (Alpha = 0.904; Part 1 = 0.803, Part 2 = 0.853; Spearman–Brown = 0.903 and 853; Guttman = 0.862; omega index = 0.84). Sample items include item 1 (I get excited about going to class =; item 36 (I get bored); item 75 (I feel so hopeless—all my energy is depleted).
2) *Achievement emotions pertaining to study* (de la Fuente, 2015b). The results showed adequate psychometric properties and acceptable fit indices. The confirmatory model showed a good fit [chi-square = 729,890; degrees of freedom (44–25) = 19; *p* < 0.001; NFI = 0.964; RFI = 0.957; IFI = 0.973; TLI = 0.978, CFI = 0.971; RMSEA = 0.080; HOELTER = 165 (*p* < *0.0*5) and 178 (*p* < *0.0*1)]. The total scale also showed good internal consistency (alpha = 0.939; Part 1 = 0.880, Part 2 = 0.864; Spearman–Brown = 0.913 and 884; Guttman = 0.903; omega index = 0.87). Sample items include item 90 (I get angry when I have to study); item 113 (My sense of confidence motivates me); item 144 (I'm proud of myself).
3) *Achievement emotions pertaining to testing* (de la Fuente, 2015c). The results showed adequate psychometric properties and acceptable fit indices. The confirmatory model showed a good fit [chi-square = 376,658; degrees of freedom (44–25) = 19; *p* < *0.0*01; NFI = 0.978; RFI = 0.969; IFI = 0.983; TLI = 0.978, CFI = 0.963; RMSEA = 0.080; HOELTER = 169 (*p* < *0.0*5) and 188 (*p* < *0.0*1). Internal consistency for the total scale was good [alpha = 0.913; Part 1 = 0.870, Part 2 = 0.864; Spearman–Brown = 0.824 and 0.869; Guttman = 0.868; omega index = 0.88]. Sample items include item 170 (Before the exam, I feel nervous and uneasy); item 181 (I enjoy taking the exam); item 224 (I am very satisfied with myself).

*Academic behavioral confidence* (an attitudinal factor) was measured by the *academic behavioral confidence scale* (Sander and Sanders, 2006, 2009) in its validated Spanish version (Sander et al., 2011). The ABC scale was developed from and tentatively positioned against the established constructs of self-concept and self-efficacy. This psychometric scale is a self-report for undergraduate students from Spain and the UK, assessing their anticipated study-related behaviors (in a program assumed to consist largely of lecture-based courses). Four subscales comprise the total ABC scale and draw out crucially distinct aspects of the academic behavior of students: grades, studying, verbalizing, and attendance (Sander, 2009). Students respond to a question stem (“How confident are you that you will be able to.”) for items, such as “.manage your workload to meet coursework deadlines” and “.write in an appropriate academic style.” Answers are given on a five-point scale (1 = “not at all confident,” 5 = “very confident”). The higher the score, the greater the confidence of the students in using effective study skills or behaviors. Prior studies yielded a four-factor model (confidence in grade achievement, studying, attending class, and discussing course material) with adequate reliability and validity (Sander and Sanders, 2009). The confirmatory model showed a good fit [chi-square = 767,516; degrees of freedom (152–54) = 98; a probability level = 0.000; NFI = 0.969; RFI = 0.962; IFI = 0.973; TLI = 0.967, CFI = 0.973; RMSEA = 0.073; HOELTER = 203 (*p* < *0.0*5) and 222 (*p* < *0.0*1)]. Internal consistency for the total scale was good [alpha = 0.952; Part 1 = 0.932, Part 2 = 0.872; Spearman–Brown = 0.961; Guttman = 0.935; omega index = 0.87].

*Engagement* (a motivational factor). For this variable, we used a validated Spanish version of the *Utrecht Work Engagement-Burnout Scale* (Schaufeli et al., 2002; Schaufeli and Bakker, 2003). The psychometric properties were satisfactory with a sample of students from Spain. The model obtained good fit indices, showing a second-order structure of three factors: vigor, dedication, and absorption. Also verified were scale unidimensionality and metric invariance in the samples assessed (chi square = 792,526, df = 74, *p* < *0.0*01; CFI = 0.954, TLI = 0.976, IFI = 0.954, TLI = 0.979, and CFI = 0.923; RMSEA = 0.083; HOELTER = 153, *p* < *0.0*5; 170 *p* < *0.0*1). The Cronbach alpha for this sample was 0.900 (14 items); 0.856 (7 items) and 0.786 (7 items) for the two parts, respectively; omega index = 0.85.

*Burnout* (a motivational factor). The validated Spanish version of the *engagement-burnout scale* (Schaufeli et al., 2002) was used. The psychometric properties with a sample of students from Spain were satisfactory. Good fit indices were obtained, showing a second-order structure of three factors: exhaustion or depletion, cynicism, and lack of effectiveness. Also verified were scale unidimensionality and metric invariance in the samples assessed [chi square = 767.885, *df* = 87, *p* < *0.0*01; CFI = 0.956, TLI = 0.964, IFI = 0.951, TLI = 0.951, and CFI = 0.953; RMSEA = 0.071; HOELTER = 224, *p* < *0.0*5; 246 *p* < *0.0*1]. The Cronbach alpha for this sample was 0.874 (15 items); 0.853 (8 items) and 0.793 (7 items) for the two parts, respectively; omega index = 0.88.

##### Teaching Variables

*Regulatory teaching (a meta-instructional variable)*. The student version of the assessment of the teaching-learning process (ATLP) (de la Fuente et al., 2012) was used to evaluate how students perceive the teaching process. The scale that addresses regulatory teaching constitutes Dimension 1 of the confirmatory model. The ATLP-D1 contains 29 items with a five-factor structure: specific regulatory teaching, regulatory assessment, preparation for learning, satisfaction with the teaching, and general regulatory teaching. Having been previously validated in university students (de la Fuente et al., 2012), the scale shows a factor structure with adequate fit indices (chi-square = 590.626; df = 48, *p* <0.001, CF1 = 0.838, TLI = 0.839, NFI = 0.850, NNFI = 0.867; RMSEA = 0.068). Internal consistency is also adequate (ATLP D1: a = 0.83; specific regulatory teaching, a = 0.897; regulatory assessment, a = 0.883; preparation for learning, a = 0.849; satisfaction with the teaching, a = 0.883, and general regulatory teaching, a = 0.883); omega index = 0.80.

*Factors of stress. Cuestionario de Estrés Académico* (*CEA*) [Academic factors of a stress questionnaire] (Cabanach et al., 2008, 2016). In order to analyze the internal structure of the scale, we conducted a confirmatory factor analysis (CFA) of the whole set of data from our sample and thus verified the second-level structure. The default model has a good fit [chi-square = 66,457, df = 13, *p* < 0.001; CFI = 0.935, TLI = 0.961, IFI = 0.947, RFI = 0.965, and NFI = 0.947; RMSEA = 0.057; HOELTER = 0.430 (*p* < 0.05) and 0.532 (*p* < 0.01)]. The proposed model contains 53 items with a seven-factor structure and two dimensions, where one factor differs from the original version. The resulting factors were (1) *stress in learning dimension*: task overload (Factor 2), difficulty performance control (F3), social climate (Factor 5), and test anxiety (Factor 7); (2) *Stress in teaching dimension*: methodology difficulties (Factor 1), public interventions (Factor 4); content lacks value (Factor 6). Overall reliability = 0.961; part 1 = 0.932, part 2 = 0.946; omega index = 0.88.

##### Product Factors

*Effects of Stress. Stress response questionnaire* (*CRE*) (Cabanach et al., 2007). We found adequate psychometric properties for this scale in this sample of Spanish students. The confirmatory structural model of the CRE has the following dimensions [Chi-square = 846.503; Degrees of freedom (275–76) = 199, *p* < 0.001; NFI = 0.952; RFI = 0.965; IFI = 0.953): F1. Burnout; F2. Sleep difficulties; F3. Irritability; F4. Negative thoughts; F5. Agitation. Scale unidimensionality and metric invariance in the samples were confirmed [RMSEA = 0.046; CFI.922 and TLI 0.901; HOELTER = 431 (*p* < 0.05) and 459 (*p* < 0.01)]. Cronbach's alpha was 0.920, part 1 = 0.874 and part 2 = 0.863; omega index = 0.90.

### Procedure

The students were informed about the research, and the volunteers completed the online self-informed consent on the *e-Coping with Stress Platform* (de la Fuente, 2015b) [http://www.inetas.net]. The questionnaires were then completed outside of normal class hours. They were asked to complete the questionnaires over one semester, during the period of September 2019 to February 2020. They completed one of the questionnaires each weekend in the order in which they were presented in the description; each questionnaire was completed one time during a 13-week period. A *Certificate of Participation in R & D Project* (10 h) was awarded, acknowledging the number of participation hours.

The platform organized the data anonymously, assigning a number to each user, whereby the different completed inventories were associated accordingly. The R and D Project was approved by the ethics committee of the University of Navarra (Ref. 2018.170).

### Data Analysis

An *ex post facto*, transversal design of linear analysis was used to test the hypotheses.

#### Preliminary Analyses: Normality Assumptions

First, we explored the quality of the data by testing for outliers and missing cases. We tested for *univariate* outliers by calculating the typical scores for each variable, considering cases with Z scores outside the +/−3 range to be potentially atypical cases (Tabachnick and Fidell, 2001a,b). In addition, the Mahalanobis distance (D2) was used to detect atypical *combinations* of variables (atypical multivariate cases), a statistical measure of multidimensional distance of an individual from the centroid or mean of the given observations (Lohr, 1999). This procedure detects significant distances from the typical combinations or centroids of a set of variables. The literature suggests removing univariate and multivariate outliers, or reassigning them the nearest extreme score (Weston and Gore, 2006). The procedure was carried out, using SPSS (v.26, IBM, Armonk, NY, USA), which includes a specific routine for missing values analysis that determines the magnitude of missing values and whether they are presented in a systematic or random manner.

#### Linear Association

We conducted bivariate correlational analyses (Pearson, two-tailed) with the total factor scores for the construct of the model. IBM-SPSS v. 25 was used for both analyses.

#### Path Analysis of Exploratory Prediction

Exploratory predictive hypotheses were tested, using path analysis with a mediational model, for multiple measurements (Ato and Vallejo, 2011). For each hypothesis posed, we tested a different empirical model of path analysis (Byrne, 2016). The first two models related to the predictive analysis of *protective factors* (buffers) against academic stress, while the second two focused on the prediction of *risk factors* for academic stress. Models 2 and 4 were selected, because they fulfilled the statistical parameters and responded empirically to the proposed integrative model. We assessed the model fit by first examining the ratio of chi-square to degrees of freedom, then the comparative fit index (CFI), normed fit index (NFI), incremental fit index (IFI), and relative fit index (RFI). All fit measures of the incremental model were above the suggested limit of 0.90 (Bentler, 1990): Comparative fit index (CFI), incremental fit index (IFI), normed fit index (NFI), relative fit index (RFI), and Tucker–Lewis index (TLI). We replicated the results of the original scale. The value of the root mean square error of approximation (RMSEA) was 0.084, less than the warning value of 0.09 (Vázquez et al., 2006). We also used the Hoelter index to determine the adequacy of the sample size. AMOS (v.22) was used for these analyses. Keith (2006) proposed the following beta coefficients as research benchmarks for *direct effects*: < 0.05 is considered too small to be meaningful, above.05 is small but meaningful, above.10 is moderate, and above.25 is large. For *indirect effects*, we used the definition of an indirect effect as the product of two effects; using Keith's benchmarks above, we propose a small indirect effect =0.003, moderate =0.01, and large =0.06, values that are significant in the sphere of education.

## Results

### Preliminary Analyses: Normality Assumptions

The descriptive and normality results showed the fit required for using linear analyses with the variables of the sample. Regarding asymmetry and kurtosis, in most cases, the obtained values were <0.500. As for the Kolmogorov–Smirnoff test, the distribution of values was not significantly different from a normal distribution. See Table 1.

### Correlations

#### Protective Factors Against Academic Stress

The personal (presage) factors of *conscientiousness* and *positivity* were found in statistically significant association with different constituent factors of the competency for studying, learning, and performing under stress (process). These characteristic factors of the SLPS competency were also significantly associated among themselves, namely, *deep approach, self-regulation, problem-focused coping, resilience, positive achievement emotions, engagement*, and *academic behavioral confidence*. These factors, in turn, were negatively associated with stress responses (product), defined as *stress factors of the learning process*, and *stress symptoms*. In complementary fashion, *Regulatory teaching* (presage) was associated positively with certain constituent factors of the SLPS competency and also negatively with *stress factors of the teaching process* (process). See Table 2.

**Table 2:** Distribution and normalization statistics of the sample (*n* = 564).

**Variables**	**Range**	**Min**	**Max**	**Med**	**(sd)**	**Asym**	**Kurtosis**	**Kolmogorov-Smirnoff**
CONSC	1–5	1.83	5.00	3.69	(0.57)	−0.260	0.015	0.192^*^
NEUROT	1–5	1.00	5.00	2.65	(0.74)	0.225	0.003	0.110^*^
POSIT	1–5	1.25	5.00	3.76	(0.67)	−0.540	0.403	0.118^*^
DEEP.LEARN.	1–5	1.00	5.00	2.96	(0.16)	−0.079	0.032	0.115^*^
SURF.LEARN.	1–5	1.00	5.00	2.16	(0.19)	0.572	0.588	0.116^*^
SR	1–5	1.21	5.00	3.48	(0.60)	−0.182	−0.157	0.098^*^
PROCRAST	1–5	1.00	4.06	2.29	(0.65)	0.209	−0.372	0.200^*^
PROBLC	1–4	1.30	3.95	2.99	(0.41)	−0.342	0.041	0.114^*^
EMOTC	1–4	1.60	3.97	2.59	(0.30)	0.156	0.318	0.092^*^
RESIL	1–5	1.82	4.86	3.74	(0.46)	−0.466	0.421	0.200^*^
EMOTP	1–5	1.15	4.93	3.34	(0.62)	−0.130	0.260	0.200^*^
EMOTN	1–5	1.06	4.10	2.23	(0.56)	0.476	−0.075	0.200^*^
CONFIDENCE	1–5	1.00	4.86	3.74	(0.56)	−0.168	−0.016	0.200^*^
ENGAG	1–5	1.00	5.00	3.47	(0.66)	−0.215	0.302	0.200^*^
BURN	1–5	1.00	4.78	2.22	(0.17)	0.583	0.018	0.098^*^
RT	1–5	1.12	5.00	3.68	(0.63)	−0.353	0.058	0.080^*^
TEACH.STRESS	1–5	1.00	5.00	2.31	(0.71)	0.592	0.033	0.200^*^
LEARN.STRESS	1–5	1.00	4.70	2.61	(0.75)	0.114	−0.485	0.200^*^
SYMPTOM.STRESS	1–5	1.00	5.00	2.31	(0.71)	0.592	0.366	0.132^*^

*CONSC, conscientiousness; NEURO, neuroticism; POSIT, positivity; DEEP.LEARN, deep learning approach; SURF.LEARN., surface learning approach; CONFIDENCE, academic behavioral confidence; SR, self-regulation; PROCRAST, procrastination; PROBLC, problem-focused coping; EMOTC, emotion-focused coping; RESIL, resilience; EMOTP, positive achievement emotions; EMOTN, negative achievement emotions; ENGAG, engagement; BURN, burnout; RT, regulatory teaching; TEACH.STRESS, stress factors of teaching process; LEARN.STRESS, stress factors of learning process; SYMPTOM.STRESS, symptoms of stress.*

* *non-significant statistical differences with the distribution*.

#### Risk Factors of Academic Stress

The personal factor *neuroticism* had a statistically significant, negative association with *positivity* (a presage factor). Also, neuroticism had a statistically significant, positive association with different factors representing a lack of the SLPS competency for learning under stress (process), such as procrastination, burnout, and negative achievement emotions. Factors showing a lack of SLPS competency were also positively associated with one another, such as *surface approach, emotion-focused coping, negative achievement emotions*, and *burnout*, and were negatively associated with *protective factors*, such as *academic behavioral confidence, self-regulation*, and *resilience*. Such risk factors were, in turn, positively associated with stress responses (product), defined as *stress factors of the learning process* and *stress symptoms*. In a complementary fashion, *regulatory teaching* (presage) was associated positively with certain constituent factors of the SLPS competency and also negatively with *stress factors of the teaching process* (process). See Table 3.

**Table 3:** Bivariate correlations between *protective factors* against academic stress in this research (*n* = 564).

	**CONSC**	**POSIT**	**DEEP**	**SR**	**PROBC**	**RESIL**	**EMOTP**	**ENGAG**	**CONF**	**RT**	**STRTE**	**STRLE**
CONSC												
POSIT	0.435^**^											
DEEP	0.518^**^	0.371^**^										
SR	0.632^**^	0.476^**^	0.371^**^									
PROBC	0.381^**^	0.384^**^	0.293^**^	0.341^**^								
RESIL	0.455^**^	0.500^**^	0.271^**^	0.482^**^	0.414^**^							
EMOTP	0.633^**^	0.603^**^	0.593^**^	0.551^**^	0.461^**^	0.428^**^						
ENGAG	0.563^**^	0.407^**^	0.487^**^	0.450^**^	0.356^**^	0.407^**^	0.711^**^					
CONF	0.465^**^	0.264^**^	0.478^**^	0.534^**^	0.341^**^	0.500^**^	0.603^**^	0.436^**^				
RT	0.320^**^	0.307^**^	0.270^**^	0.226^**^	−0.226^**^	0.236^**^	0.352^**^	0.348^**^	0.307^**^			
STRTE	−0.118^**^	−0.342^**^	−0.142^**^		−0.063	−0.168^**^	−0.314^**^	−0.219^**^	−0.367^**^	−0.105^**^		
STRLE	−0.194^**^	−0.178^**^	−0.095^*^	−0.341^**^	−0.028	−0.106^*^	−0.285^**^	−0.221^**^	−0.178^**^	−0.34	0.614^**^	
STRSY	−0.280^**^	−0.148^**^	−0.022	−0.161^**^	−0.098^**^	−0.169^**^	−0.353^**^	−0.099^**^	−0.148^**^	−0.91^**^	0.442^**^	0.603^**^

*CONSC, conscientiousness; POSIT, positivity; DEEP, deep approach; CONF, academic behavioral confidence; SR, self-regulation; PROBC, problem-focused coping; ENGAG, engagement; EMOTP, positive achievement emotions; RESIL, resilience; RT, regulatory teaching; STRTE, stress factors of teaching process; STRLE, stress factors of learning process; STRSY, symptoms of stress.*

* *p < 0.05;*

** *p < 0.01;*

^^**^*^p < 0.001.

### Path Analysis Predictive Relationships

The four exploratory models that were tested fulfilled the statistical parameters required for the empirical fit (see Table 3). From these four models, models 2 and 4 were selected. Despite having somewhat less significance, they showed a better fit to the theoretical model on which this research is based. Model 1 shows statistics of the stress protection factors that refer exclusively to the learning process. Model 2 shows the statistics when teaching process variables are also included; for this reason, it is more powerful, and the statistical values are better fitted. Model 3 refers to risk factors for stress that are triggered by the learning process, while Model 4 incorporates stress factors from the teaching process as well, also showing better a statistical fit (see Table 4). We, therefore, present below the specific, predictive statistical values from Models 2 and 4.

**Table 4:** Bivariate correlations between *risk factors* of academic stress in this research (*n* = 564).

	**NEUROT**	**POSIT**	**SURFACE**	**SR**	**PROCR**	**EMOTC**	**RESIL**	**EMOTN**	**BURN**	**CONF**	**RT**	**STRTE**	**STRLE**
NEUROT													
POSIT	−0.325^**^												
SURFACE	0.188^**^	−0.173^**^											
SR	−0.367^**^	0.472^**^	−0.360^**^										
PROCR	0.252^**^	−0.173^**^	0.323^**^	−0.418^**^									
EMOTC	0.288^**^	−0.028	0.161^**^	−0.108^**^	0.280^**^								
RESIL	−0.280^**^	0.592^**^	−0.170^**^	0.490^**^	−0.223^**^	0.076^*^							
EMOTN	0.472^**^	−0.300^**^	0.455^**^	−0.479^**^	0.513^**^	0.263^**^	−0.315^**^						
BURNOUT	0.343^**^	−0.458^**^	0.368^**^	0.491^**^	0.452^**^	0.140^**^	−0.372^**^	592^**^					
CONF	−0.243^**^	0.377^**^	−0.318^**^	0.532^**^	−0.327^**^	0.012	0.498^**^	−0.478^**^	−0.449^**^				
RT	−0.010	0.307^**^	−0.134^**^	0.229^**^	−0.213^**^	0.083^**^	0.242^**^	−0.186^**^	−0.270^**^	0.283^**^			
STRTE	0.388^**^	−0.342^**^	0.348^**^	−0.317^**^	0.325^**^	0.174^**^	−0.229^**^	0.583^**^	0.357^**^	−0.389^**^	−0.105^*^		
STRLE	0.418^**^	−0.178^**^	0.250^**^	−0.351^**^	0.383^**^	0.315^**^	−0.149^*^	0.612^**^	0.413^**^	−0.204^**^	−0.34	0.612^**^	
STRSY	0.569^**^	−0.148^**^	0.273^**^	−0.411^**^	0.341^**^	0.342^**^	−0.233^**^	0.583^**^	0.357^**^	−0.389^**^	−0.084^*^	0.442^**^	0.603^**^

*NEUROT, neuroticism; POSIT, positivity; SURFACE, surface approach; CONF, academic behavioral confidence; SR, self-regulation; PROCR, procrastination; EMOTC, emotion-focused coping; RESIL, resilience; EMOTN, negative achievement emotions; BURNOUT, burnout; RT, regulatory teaching; STRTE, stress factors of teaching process; STRLE, stress factors of learning process; STRSY, symptoms of stress.*

* *p < 0.05;*

** *p < 0.01;*

^***^p < 0.001.

### The Model of Protective Factors Against Academic Stress (Model 2)

#### Direct Effects of the Protective Factors

The predictive structural Model 2 demonstrated many predictive relationships pertaining to students. Two *personal factors* (presage) were found to protect against stress: *conscientiousness* and *positivity*. The former significantly predicted the latter (*B* = 0.376). Both were significant predictors of different levels of the SLPS competency components (presage). Conscientiousness, as an “executive” personality variable, appeared as a significant predictor of meta-cognitive variables (deep approach; *B* = 0.190), meta-behavioral variables (self-regulation; *B* = 0.438), meta-emotional variables (problem-focused coping; *B* = 0.110), meta-motivational variables (resilience; *B* = 0.090), and meta-affective variables (academic behavioral confidence; *B* = 0.180), and also of emotional variables (positive emotions; *B* = 0.247). *Positivity*, as a personal psychological variable, appeared as a significant predictor of emotional variables (positive emotions; *B* = 0.177).

The relationships between the factors of the SLPS competency model (process) were also very significant. Thus, *deep learning* predicted academic behavioral confidence (*B* = 0.111). Academic behavioral confidence was predicted by *self-regulation* (*B* = 0.121) and *engagement* (*B* = 0.120). Self-Regulation predicted *academic behavioral confidence* (*B* = 0.177), *resilience* (*B* = 0.186), *engagement* and *positive emotions (B* = 0.465*). One* especially important predictive effect was to the predictive power of *positive emotions* with respect to *deep approach (B* = 0.450), *academic behavioral confidence* (*B* = *0.2*28), *self-regulation* (*beta* = 0.219), *problem-focused coping* (*B* = 0.280), and *resilience* (*B* = 0.118).

In the analysis of teaching process factors, the positive predictive value of *regulatory teaching* (RT) was demonstrated in regard to *positivity* (*B* = 0.11), *academic behavioral confidence (B* = 0.068*), engagement* (*B* = *0.207*), and *positive emotions* (*B* = 134). Inversely, *regulatory teaching* negatively predicted the *stress in teaching* factor (*B* = −0.115), which, in turn, negatively predicted *positive emotions* (*B* = −0.127), and positively predicted *stress in learning (B* = 0.589).

Different relations were also shown in regard to the prediction of stress symptoms, such as *stress during learning* and *stress symptoms* (product). Some factors were significant negative predictors of *stress in learning*, such as *self-regulation* (*B* = −0.171), and *positive emotions* (*B* = −0.175), but others were positive predictors, like problem-focused coping (*B* = 0.069) and, above all, *stress in teaching* (B =0.589). Finally, *stress symptoms* were negatively predicted by personal factors like *self-regulation* (*B* = −0.167) and *resilience* (*B* = −0.143), but also by *stress in learning* (*B* = *0.5*43), a factor that was already shown to be predicted by *stress in teaching*.

#### Indirect Effects of the Protective Factors

The indirect effects showed that some variables had adequate values as mediating predictors. Thus, the variables *conscientiousness* and *positivity* (presage factors) showed a positive effect on nearly all the variables belonging to the SLPS competency—*conscientiousness* having greater predictive strength—and a significant negative predictive effect on the two variables of experiencing academic stress (*B* = −0.211 and *B* = −0.119, respectively).

Second, a group of factors belonging to the SLPS competency (process factors) showed positive, predictive indirect effects among themselves (self-regulation, problem-focused coping, engagement, and positive emotions), and negative effects on *academic stress* (*B* = −0.003; *B* = −0.119; *B* = −0.037; *B* = −0.032; *B* = −0.070). Likewise, *regulatory teaching* had a positive indirect effect on most variables of the SLPS competency (confidence, *B* = *0.1*04; self-regulation, *B* = 0.078; problem-focused coping, *B* = *0.1*01; engagement, *B* = *0.0*18; positive emotions, *B* = 0.139; resilience, *B* = 0.097).

Finally, most of the SLPS variables had a negative indirect predictive effect on *academic stress* (confidence, *B* = −0.003; self-regulation, *B* = −0.019; engagement, *B* = −0.032; positive emotions, *B* = −0.037; resilience, *B* = 0.097), as did regulatory teaching (*B* = −0.066). However, positive indirect prediction factors did appear (problem-focused coping, *B* = 0.101; stress in teaching, *B* = *0.3*23). See Table 5 and Figure 1.

**Table 5:** Statistical parameters of structural models.

**Models**	**Type of factors**	**Direction**	**Chi- square**	**Degrees of freedom**	***p < ***	**RSMR**	**TLI**	**RFI**	**IFI**	**TLI**	**CFI**	***RMSEA***	***HO0.05***	***HO0.01***
Model 1	L	Protective factors	184.714	(77–51):26	0.001	0.072	0.948	0.969	0.955	0.958	0.955	0.061	349	409
Model 2	L & T	Protective factors^*^	414.536	(104–61): 43	0.001	0.051	0.958	0.987	0.969	0.969	0.954	0.066	337	370
Model 3	L	Risk factors	327.258	(90–77): 33	0.001	0.082	0.955	0.952	0.965	0.971	0.953	0.073	240	278
Model 4	L & T	Risk factors^*^	519.634	(119–69): 50	0.001	0.032	0.958	0.976	0.959	0.975	0.968	0.065	0.315	0.343

*L, learning process; T, teaching process;*

* *selected models*.

#### Combination Factors

Many total effects were combined effects of direct and indirect prediction effects. Table 5 presents the total, direct, and indirect effects (full and partial mediation effects) of protective factors of students against academic stress. Observe the predictive value of both personal characteristics and teaching process characteristics.

### A Model of Risk Factors in Academic Stress (Model 4)

#### Direct Effects of the Risk Factors

This model gave evidence of two personal factors with predictive weight (*presage factors*). *Neuroticism* had a negative predictive value for *positivity (B* = −0.304). Moreover, *neuroticism* showed a positive predictive value for several risk factors that characterize a lack of SLPS competency, such as *surface approach* (*B* = 0.208), *procrastination* (*B* = 0.331), *burnout* (*B* = 0.102) and *negative emotions* (*B* = 0.180). It also showed a negative predictive value on protective factors like *academic behavioral confidence* (*B* = −0.231) and *self-regulation* (*B* = −0.188).

When analyzing the risk factors belonging to the SLPS competency model, we confirmed significant predictive relationships between them. *Surface approach* negatively predicted *academic behavioral confidence* (*B* = −0.164) and *self-regulation* (*B* = −0.266), and positively predicted *negative emotions* (*B* = 0.243). *Procrastination* negatively predicted self-regulation (*B* = −0.129) and positively predicted *emotion-focused coping* (*B* = *0.1*70), *burnout* (*B* =*0.2*08) and *negative emotions* (*B* = *0.2*37). Also, *emotion-focused coping* and *burnout* predicted *negative emotions* (*B* = *0.1*60; *B* = *0.1*96, respectively).

As for context factors, *regulatory teaching* appeared as a protective factor that, in addition to positively predicting protective factors like *positivity* (*B* = 0.220), *academic behavioral confidence* (*B* = 0.144) and *self-regulation* (*B* = 0.107), it negatively predicted risk factors like *procrastination* (*B* = −0.111), *burnout* (*B* = −0.116) and *stress in teaching* (*B* = −0.100). However, the risk factor *stress in teaching* positively predicted *stress in learning* (*B* = 0.391).

Finally, the risk factors of the SLPS competency predicted experiences of academic stress. *Negative emotions* predicted *stress in learning* (*B* = 0.163). *Procrastination* (*B* = 0.185) and *stress in teaching* (*B* = 0.391), as risk factors, positively predicted *stress symptoms. Stress in learning* positively predicted *stress symptoms* (*B* =*0.4*87). By contrast, protective factors like *self-regulation* (*B* = −0.142) and *resilience* (*B* = −0.131) negatively predicted stress experiences.

#### Indirect Effects of the Risk Factors

*Neuroticism*, as a personal risk factor (presage), showed numerous indirect effects that positively predicted risk factors of the SLPS competency, such as *emotion-focused coping* (*B* = *0.0*57), *burnout* (*B* = *0.2*31) and *negative emotions* (*B* = 0.239). In addition, its indirect effects negatively predicted protective factors like *academic behavioral confidence* (*B* = −0.238), *self-regulation* (*B* = −0.189) and *resilience* (*B* = −0.267).

Regarding risk factors of the SLPS competency (*process factors*), certain risk factors (*surface approach* and *procrastination*) showed the indirect effect of positively predicting other risk factors (*emotion-focused coping, burnout*, and *negative emotions)*. The opposite occurred with personal protective factors (*academic behavioral confidence* and *self-regulation*) and contextual protective factors (*regulatory teaching*), where risk factors were negatively predicted (*burnout* and *negative emotions*).

**Figure 3:**
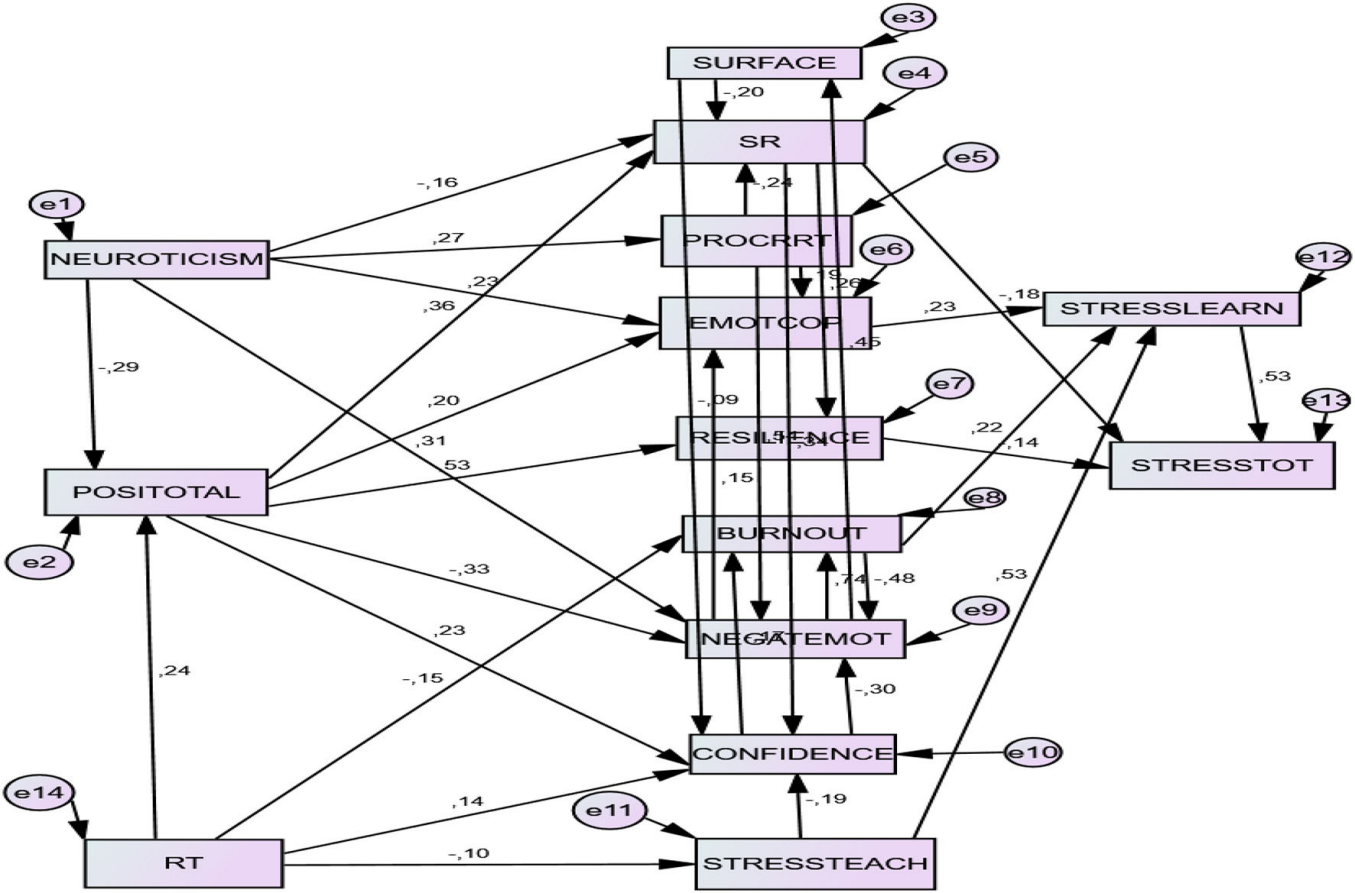
A structural predictive model of risk factors for academic stress (Model 4). POSITOTAL, positivity; SURFACE, surface approach; CONFIDENCE, academic behavioral confidence; SR, self-regulation; PROCRRT, procrastination; EMOTCOP, emotion-focused coping; NEGATEMOT, negative achievement emotions; RT, regulatory teaching; STRESSTEACH, stress factors of teaching process; STRESSLEARN, stress factors of learning process; STRESSTOT, symptoms of stress; CI, confidence interval. Bootstrapping sample size = 430.

Finally, there was an indirect effect that positively predicted *stress symptoms* (*product* factor): from *neuroticism* (a *presage* factor), *surface approach, procrastination, burnout, negative emotions*, and *stress in teaching* (*process* factors). Protective factors like *positivity* (*presage* factor), *academic behavioral confidence, self-regulation, resilience*, and *regulatory teaching* (*process* factors) appeared as negative predictors of *stress symptoms* (a *product* factor). See Table 6 and Figure 3.

**Table 6:** Total, indirect, and direct effects of *stress protection factors* in this study, and 95% bootstrap confidence intervals (CI).

**Predictive variable**	**Criterion variable**	**Total effect**	**CI (95%)**	**Direct effect**	**CI (95%)**	**Indirect effect**	**CI (95%)**	**Results, effects**	**CI (95%)**
CONSC–>	Positivity	0.376	[0.32, 0.43]	0.376	[0.32, 0.43]	0.00	[−0.03, 0.04]	Direct only	[0.32, 0.43]
CONSC—>	Deep Appr	0.440	[0.48, 0.39]	0.190	[0.16, 0.23]	0.248	[0.20, 0.28]	Partial mediat	[0.20, 0.28]
CONSC–>	Self-Regul	0.621	[0.59, 0.72]	0.438	[0.41, 0.56]	0.183	[0.11, 0.25]	Partial mediat	[0.11, 0.25]
CONSC–>	Problem coping	0.348	[0.29, 0.40]	0.110	[0.06, 0.16]	0.238	[0.18, 0.29]	Partial mediat	[0.18, 0.29]
CONSC–>	Resilience	0.444	[0.41, 0.48]	0.090	[0.02, 0.14]	0.354	[0.29, 0.39]	Full mediation	[0.39, 0.29]
CONSC–>	Posit. emot	0.551	[0.50, 0.61]	0.247	[0.16, 0.32]	0.304	[0.20, 0.40]	Partial mediat	[0.20, 0.40]
CONSC–>	Acad. confid	0.513	[0.62, 0.40]	0.180	[0.12, 0.23]	0.333	[0.29, 0.39]	Partial mediat	[0.29, 0.39]
CONSC–>	Engagement	0.511	[0.39, 0.62]	0.458	[0.34, 0.56]	0.053	[0.01, 0.09]	Direct only	[0.34, 0.56]
CONSC–>	Regul. Teach	0.00		0.00		0.00		Non-effect	
CONSC–>	Stress Teach	0.00		0.00		0.00		Non-effect	
CONSC–>	Stress Learn	−0.083	[−0.02, 13]	0.00	[−0.04, 0.07]	−0.083	[−0.02, 13]	Full mediation	[−0.02, 13]
CONSC–>	Stress Sympt	−0.211	[−0.17, −0.24]	0.00	[−0.05, 0.08]	−0.211	[−0.17, −0.24]	Full mediation	[−0.17, −0.24]
Positivity–>	Deep Appr	0.084	[0.02, 15]	0.00	[−0.03, 05]	0.084	[0.02, 15]	Partial mediat	[0.02, 15]
Positivity–>	Self-Regul	0.041	[0.01, 0.09]	0.00	[−0.01, 04]	0.041	[0.01, 0.09]	Partial mediat	[0.01, 0.09]
Positivity–>	Probl. coping	0.053	[−0.03, 0.11]	0.00	[−0.03, 07]	0.053	[−0.03, 0.011]	Partial mediat	[−0.03, 0.011]
Positivity–>	Resilience	0.061	[0.01, 0.11]	0.00	[−0.04, 09]	0.061	[0.01, 0.11]	Partial mediat	[0.01, 0.11]
Positivity–>	Posit. emot	0.177	[0.10, 0.26]	0.177	[0.10, 0.26]	0.00	[−0.04, 0.06]	Direct only	[0.10, 0.26]
Positivity->	Acad. confi	0.089	[0.01, 14]	0.00	[−0.03, 0.05]	0.089	[0.01, 14]	Only indirect	[0.01, 14]
Positivity->	Engagement	0.023	[−0.02, 06]	0.00	[−0.06, 0.07]	0.023	[−0.02, 06]	Only indirect	[−0.02, 06]
Positivity->	Regul teach	0.00		0.00		0.00		Non-effect	
Positivity->	Stress Teach	0.00		0.00		0.00		Non-effect	
Positivity->	Stress Learn	0.00		0.00		0.00		Non-effect	
Positivity->	Stress Sympt	−0.117	[−0.19, −0.05]	0.00	[−0.04, 0.07]	−0.117	[−0.19, −0.05]	Only indirect	[−0.19, −0.05]
Deep Learning–>	Self-Regul.	0.001		0.00		0.001	[−0.010, −0.005]	Only indirect	[−0.010, −0.005]
Deep Learning–>	Probl. coping	0.053	[−0.07, −0.03]	0.00	[−0.03, 0.05]	0.053	[−0.07, −0.03]	Only indirect	[−0.07, −0.03]
Deep Learning–>	Resilience	0.001	[−0.09, −0.003]	0.00	[−0.06, 0.09]	0.001	[−0.09, −0.003]	Only indirect	[−0.09, −0.003]
Deep Learning–>	Posit. emot	0.005	[0.23, 0.78]	0.00	[−0.04, 0.07]	0.005	[0.001, 0.008]	Only direct	[0.23, 0.78]
Deep Learning—>	Acad. confi	0.113	[0.08, 0.14]	0.111	[0.08, 0.14]	0.002	[0.001, 0.007]	Only indirect	[0.08, 0.14]
Deep Learning–>	Engagement	0.012	[0.06, 0.015]	0.00	[−0.03, 0.05]	0.012	[0.06, 0.015]	Only indirect	[0.06, 0.015]
Deep Learning–>	Regul teach							Non-effect	
Deep Learning–>	Stress Teach							Non-effect	
Deep Learning–>	Stress Learn	−0.017	[−0.012, −0.026]	0.00	[−0.04, 0.07]	−0.017	[−0.012, −0.026]	Only indirect	[−0.012, −0.026
Deep Learning–>	Stress Sympt							Non-effects	
Self-regulation–>	Probl coping	0.002	[−0.001, 0.006]	0.00	[−0.06, 0.04]	0.002	[−0.001, 0.006]	Only indirect	[−0.001, 0.006]
Self-regulation–>	Resilience	0.188	[0.154, 0.213]	0.186	[0.154, 0.213]	0.002	[−0.001, 0.005]	Only direct	[0.154, 0.213]
Self-regulation–>	Posit. emot	0.228	[0.110, 0.267]	0.219	[0.111, 0.258]	0.009	[0.001, 0.018]	Only direct	[0.111, 0.258]
Self-regulation–>	Acad. confi	0.180	[0.201, 0.165]	0.177	[0.182, 163]	0.003	[−0.001, 0.004]	Only direct	[0.182, 163]
Self-regulation–>	Engagement	0.019	[0.09, 0.26]	0.00	[−0.05, 0.08]	0.019	[0.09, 0.26]	Full mediation	[0.09, 0.26]
Self-regulation–>	Regul teach							Non-effect	
Self-regulation–>	Stress Teach	−0.159	[−0.087, −0.23]	0.00	[−0.05, 0.08]	−0.159	[−0.087, −0.23]	Full mediation	[−0.087, −0.23]
Self-regulation–>	Stress Learn	−0.173	[−0.08, −0.24]	−0.173	[−0.08, −0.24]	0.00		Only direct	
Self-regulation–>	Stress Sympt	−0.288	[−0.145, −0.316]	−0.167	[−0.134, −0.186]	−0.119	[−0.090, −0.134]	Partial mediat	[−0.090, −0.134]
Problem coping–>	Resilience							Non-effect	
Problem coping–>	Posit. emot	0.280	[0.127, 0.348]	0.280	[0.127, 0.348]	0.00	[−0.03, 08]	Only direct	[0.127, 0.348]
Problem coping–>	Acad. confi	0.014	[0.03, 0.031]	0.00	[−0.06, 0.09]	0.014	[0.03, 0.031]	Full mediation	[−0.06, 0.036
Problem coping–>	Engagement							Non-effect	
Problem coping–>	Regul teach							Non-effect	
Problem coping–>	Stress Teach							Non-effect	
Problem coping–>	Stress Learn	0.069	[0.043, 0.075]	0.069	[0.043, 0.075]	0.00	[−0.04, 0.09]	Direct only	[0.043, 0.075]
Problem coping–>	Stress Sympt	0.037	[0.021, 0.048]	0.00	[−0.06, 0.07]	0.037	[0.021, 0.48]	Partial mediat	[0.021, 0.48]
Resilience–>	Posit. emot	0.161	[0.132, 0.184]	0.118	[0.07, 0.28]	0.043	[0.018, 0.056]	Partial mediat	[0.018, 0.056]
Resilience–>	Acad. confi	0.008	[0.003, 0.010]	0.00	[−0.07, 0.08]	0.008	[0.003, 0.010]	Full mediation	[0.003, 0.010]
Resilience–>	Engagement							Non-effect	
Resilience–>	Regul teach							Non-effect	
Resilience–>	Stress Teach	−0.140	[−0.11, −0.23]	0.00	[−0.033, 0.04]	−0.140	[−0.124, −0.231]	Full mediation	[−0.124, −0.231]
Resilience–>	Stress Learn							Non-effect	
Resilience–>	Stress Sympt	−0.141	[−0.10, −0.24]	−0.141	[−0.10, −0.24]	0.00	[−0.04, 0.06]	Direct only	[−0.10, −0.24]
Posit. emot–>	Deep Appr	0.450	[0.65, 23]	0.450	[0.65, 23]	0.00	[−0.06, 0.10]	Direct only	[0.65, 23]
Posit. emot–>	Acad. confi	0.228	[0.12, 0.39]	0.228	[0.12, 0.39]	0.093	[0.01, 0.016]	Partial mediat	[0.12, 0.39]
Posit. emot–>	Engagement	0.033	[0.01, 0.05]	0.00	[−0.04, 0.06]	0.033	[−0.01, 0.06]	Full mediation	[0.01, 0.05]
Posit. emot–>	Regul teach							Non-effect	
Posit. emot–>	Stress Teach							Non-effect	
Posit. emot–>	Stress Learn	−0.175	[−0.078, −0.213]	−0.175	[−0.078, −0.213]	−0.019	[−0.007, −0.034]	Partial mediat	[−0.007, −0.034]
Posit. emot—>	Stress Sympt	−0.070	[−0.050, −0.096]	0.00	[−0.04, 0.07]	−0.070	[−0.050, −0.096]	Only indirect	[−0.050, −0.096]
Academic conf—>	Self–Regul	0.132	[0.007, 0.221]	0.121	[0.10, 0.21]	0.011	[−0.003, 0.022	Only directr	[0.10, 0.21
Academic conf—>	Engagement	0.106	[0.095, 0.122]	0.104	[0.095, 0.125]	0.002	[−0.001, 0.003]	Partial mediat	[−0.001, 0.003]
Academic conf—>	Regul teach							Non-effect	
Academic conf—>	Stress Teach							Non-effect	
Academic conf—>	Stress Learn	−0.001	[−0.03, −0.005]	0.00	[−0.003, 0.006]	−0.001	[−0.03, −0.005]	Full mediation	[−0.03, −0.005]
Academic conf—>	Stress Sympt	−0.003	[−0.07, −0.004]	0.00	[−0.05, 0.07]	−0.003	[−0.07, −0.004]	Full mediation	[−0.07, −0.004]
Engagement—>	Deep Appr	0.213	[0.198, 0.314]	0.00	[−0.03, 0.08]	0.213	[0.198, 0.314]	Full mediation	[0.198, 0.314]
Engagement—>	Self–Reg	0.103	[0.086, 0.121]	0.00	[−0.01, 0.04]	0.103	[0.086, 0.121]	Full mediation	[0.086, 0.121]
Engagement—>	Probl.coping	0.132	[0.084, 0.142]	0.00	[−0.09, 0.07]	0.132	[0.084, 0.142]	Full mediation	[0.084, 0.142]
Engagement—>	Resilience	0.075	[0.053, 0.092]	0.00	[−0.04, 0.03]	0.075	[0.053, 0.092]	Full mediation	[0.053, 0.092]
Engagement—>	Posit. emot	0.465	[0.33, 0.57]	0.465	[0.33, 0.57]	0.007	[−0.002, 0.015]	Direct only	[0.33, 0.57]
Engagement—>	Acad. confi	0.149	[0.087, 0.235]	0.00	[−0.01, 0.05]	0.149	[0.087, 0.235]	Full mediation	[0.087, 0.235]
Engagement—>	Regul teach							Non-effect	
Engagement—>	Stress Teach							Non-effect	
Engagement—>	Stress Learn	−0.009	[−0.012, −0.003]	0.00	[−0.004, 0.007]	−0.009	[−0.012, −0.003]	Full mediation	[−0.012, −0.003]
Engagement—>	Stress Symt	−0.032	[−0.041, −0.018]	0.00	[−0.03, 0.07]	−0.032	[−0.041, −0.018]	Full mediation	[−0.041, −0.018]
Regul. Teach—>	Positivity	0.110	[0.08, 0.13]	0.110	[0.08, 0.13]	0.00	[−0.04, 0.05]	Direct only	[0.08, 0.13]
Regul. Teach—>	Deep Learn							Non-effect	
Regul. Teach—>	Self–Reg	0.078	[0.031, 0.093]	0.00	[−0.02, 0.06]	0.078	[0.031, 0.093]	Full mediation	[0.031, 0.093]
Regul. Teach—>	Prob.coping	0.101	[0.087, 0.145]	0.00	[−0.04, 0.07]	0.101	[0.087., 0.145]	Full mediation	[0.087., 0.145]
Regul. Teach—>	Resilience	0.097	[0.05, 0.123]	0.00	[−0.03, 0.07]	0.097	[0.05, 0.123]	Full mediation	[05, 0.123]
Regul. Teach—>	Posit. emot	0.271	[0.210, 0.321]	0.134	[0.084, 0.238]	0.139	[0.075, 0.216]	Partial mediat	[0.075, 0.216]
Regul. Teach—>	Acad. confi	0.172	[0.113, 0.195]	0.068	[0.032, 0.89]	0.104	[0.87, 0.135]	Partial mediat	[0.87, 0.135]
Regul. Teach—>	Engagement	0.225	[0.118, 0.236]	0.207	[0.196, 0.236]	0.018	[0.07, 0.26]	Partial mediat	[0.07, 0.26]
Regul. Teach—>	Stress Teach	−0.115	[−0.131, −0.108]	−0.115	[−0.131, −0.108]	0.00	[−0.02, 0.08]	Direct only	[−0.131, −0.108
Regul. Teach—>	Stress Learn	−0.074	[−0.043, 0.094]	0.00	[−0.06, 05]	−0.074	[−0.043, 0.094]	Full mediation	[−0.043, 0.094]
Regul. Teach—>	Stress Sympt	−0.066	[−0.090, −0.01]	0.00	[−0.02, 0.05]	−0.066	[−0.090, −0.01]	Full mediation	[−0.090, −0.01]
Stress Teach—>	Positivity							Non-effect	
Stress Teach—>	Deep Learn	−0.058	[−0.08, 0.02]	0.00	[−0.05, 0.03]	−0.058	[−0.08, 0.02]	Full mediation	[−0.08, 0.02]
Stress Teach—>	Self–Reg	−0.028	[−0.07, 0.03]	0.00	[0.−04, 0.08]	−0.028	[−0.07, 0.03]	Full mediation	[−0.07, 0.03]
Stress Teach—>	Prob. coping	−0.036	[−0.24, 0.67]	0.00	[−0.03, 08]	−0.036	[−0.24, 0.67]	Full mediation	
Stress Teach—>	Resilience	−0.020	[−0.034, −0.08]	0.00	[−0.03, 0.12]	−0.020	[−0.034, −0.08]	Full mediation	[−0.034, −0.08]
Stress Teach—>	Posit. emot	−0.127	[−0.132, −0.111]	−0.127	[−0.132, −0.111]	0.00	[−0.03, 0.08]	Direct only	[−0.132, −0.111]
Stress Teach—>	Acad. confi	−0.041	[−0.021, 0.054]	0.00	[−0.06, 0.07]	−0.041	[−0.021, 0.054]	Full mediation	[−0.021, 0.054]
Stress Teach—>	Engagement	−0.004	[−0.007, 0.003]	0.00	[−0.08, 0.06]	−0.004	[−0.007, 0.003]	Full mediation	[−0.007, 0.003]
Stress Teach—>	Stress Learn	0.561	[0.438, 0.649]	0.589	[0.438, 0.749]	0.002	[−0.003, 0.005]	Direct only	[0.438, 0.649]
Stress Teach—>	Stress Sympt	0.323	[0.225, 0.426]	0.00	[−0.002, 0.012]	0.323	[0.225, 0.426]	Full mediation	[0.225, 0.426]
Stress Learn—>	Stress Teach	0.529	[0.423, 0.721]	0.00	[−0.05, 0.10]	0.529	[0.423, 0.721]	Full mediation	[0.423, 0.721]
Stress Learn—>	Stress Sympt	0.534	[0.213, 0.678]	0.534	[0.213, 0.678]	0.00	[−0.021, 0.045]	Direct only	[0.213, 0.678]

*Bootstrapping sample size = 564. Model 2 (teaching and learning factors).*

*CONSC, conscientiousness; deep learning, deep approach; Acad. confi, academic behavioral confidence; Self-Reg, self-regulation; Prob. coping, problem-focused coping; Posit. emot, positive achievement emotions; Regul. Teach, regulatory teaching; Stress Teach, stress factors of teaching process; Stress Learn, stress factors of learning process; Stress Sympt, symptoms of stress; CI, confidence interval. Bootstrapping sample size = 430*.

#### Combination Factors

In this model, many total effects were also combined effects of direct and indirect prediction effects. Table 7 presents the total, direct, and indirect effects (full and partial mediation effects) of risk factors of students for academic stress. Observe the predictive value of both personal characteristics and teaching process characteristics.

**Table 7:** Total, indirect, and direct effects of *stress risk factors* in this study, and 95% bootstrap confidence intervals (CI).

**Predictive variable**	**Criterion variable**	**Total effect**	**CI (95%)**	**Direct effect**	**CI (95%)**	**Indirect effect**	**CI (95%)**	**Results, effects**	**CI (95%)**
Neuroticism—>	Positivity	−0.304	[−0.43, −0.23]	−0.304	[−0.43, −0.23]	0.00	[−0.03, 0.04]	Direct only	[−0.43, −0.23
Neuroticism—>	Surf. Learn.	0.208	[0.18, 0.29]	0.208	[0.18, 0.29]	0.00	[−0.04, 0.08]	Direct only	[0.18, 0.29]
Neuroticism—>	Self–Regulation	−0.377	[−0.54, −0.22]	−0.188	[−0.24, −0.10]	−0.189	[−0.24, −0.10]	Partial mediation	[−0.24, −0.10]
Neuroticism—>	Procrastinat	0.331	[0.121, 0.546]	0.331	[0.121, 0.546]	0.00	[−0.08, 0.012	Direct only	[0.121, 0.546]
Neuroticism—>	Emo. Coping	0.057	[0.02, 0.08]	0.00	[−0.03, 0.05]	0.057	[0.02, 0.08]	Partial mediation	[0.02, 0.08]
Neuroticism—>	Resilience	−0.267	[−0.41, −0.17]	0.00	[−0.04, 0.07]	−0.267	[−0.271, −0.232]	Full mediation	[−0.271, −0.232]
Neuroticism—>	Negat.Emot	0.419	[0.56, 0.27]	0.180	[0.12, 0.31]	0.239	[0.247, 0.223]	Partial mediation	[0.247, 0.223]
Neuroticism—>	Acad. confi	−0.476	[−0.572, −321]	−0.238	[−321, −0.212]	−0.238	[−0.241, −0.221]	Partial mediation	[−0.241, −0.221]
Neuroticism—>	Burnout	0.333	[0.442, 0.223]	0.102	[0.198, 0.08]	0.231	[0.242, 0.223]	Partial mediation	[0.242, 0.223]
Neuroticism–>	Regul. Teach	0.00		0.00		0.00		Non-effect	
Neuroticism–>	Stress Teach	0.00		0.00		0.00		Non-effect	
Neuroticism–>	Stress Learn	0.202	[0.234, 0.210]	0.00	[−0.03, 0.07]	0.202	[0.234, 0.210]	Full mediation	[0.234, 0.210]
Neuroticism–>	Symt. Stres	0.248	[0.229, 0.259]	0.00		0.248	[0.229, 0.259]	Full mediation	[0.229, 0.259]
Positivity—>	Surf. Learn	00		0.00		0.00		Partial mediat	[0.20, 0.28]
Positivity—>	Self–Regulation	0.300	[0.342, 0.232]	0.300	[0.342, 0.232]	0.00	[−0.05, 0.09]	Direct only	[0.342, 0.232]
Positivity—>	Procrastinat	0.00		0.00		0.00		Non-effect	
Positivity—>	Emot.coping	0.228	[0.325, 0.438]	0.228	[0.325, 438]	0.00	[−0.02, 0.08]	Direct Only	[0.325, 0.438]
Positivity–>	Resilience	0.523	[0.55, 0.50]	0.523	[0.55, 0.50]	0.086	[−0.92,−0.71]	Partial mediation	[−0.92,−0.71]
Positivity–>	Negat. Emot	−0.176	[−0.234, −0.156]	−0.148	[−0.159, −0.129]	−0.028	[−0.010, −0.038]	Partial mediat	[−0.010, −0.038]
Positivity–>	Acad. confi	0.350	[0.382, 0.338]	0.248	[0.267, 0.224]	0.102	[0.95, 0.134]	Partial mediat	[0.95, 0.134]
Positivity–>	Burnout	−0.141	[−0.162, −0.128]	0.00	[−0.03, 0.04]	−0.141	[−0.162, −0.128]	Full mediat	[−0.162, −0.128]
Positivity–>	Regul Teach	0.00		0.00		0.00		Non-effect	
Positivity–>	Stress Teach	0.00		0.00		0.00		Non-effect	
Positivity–>	Stress Learn	0.00	[−0.03, 0.05]	0.00	[−0.03, 0.05]	−0.034	[−0.054, −0.021]	Full mediat	[−0.054, −0.021]
Positivity–>	Stress Symt	−0.139	[−0.145, −0.121]	0.00	[−0.05, 0.06]	−0.139	[−0.145, −0.121]	Full mediat	[−0.145, −0.121]
Surf Learning—>	Self–Regulation	−0.266	[−0.24, −0.21]	−0.266	[−0.24, −0.21]	0.00	[−0.03, 0.07]	Direct only	[−0.24, −0.21]
Surf Learning—>	Procrastinat	0.00		0.00		0.00		Non-effect	0.00
Surf Learning—>	Emo. coping	0.048	[0.025, 0.065]	0.00	[−0.04, 0.07]	0.048	[0.025, 0.065]	Full mediat	[0.025, 0.065]
Surf Learning—>	Resilience	−0.076	[−0.093, −0.034]	0.00	[−0.05, 0.06]	−0.076	[−0.093, −0.034]	Full mediat	[−0.093, −0.034]
Surf Learning—>	Negat. Emot	0.243	[0.27, 0.35]	0.243	[0.27, 0.35]	0.054	[0.012, 0.087]	Partial mediat	[0.012, 0.087]
Surf Learning—>	Acad. confi	−0.254	[−0.351, −0.125]	−0.164	[−0.267, −0.086]	−0.090	[−056. −0.134]	Partial mediat	[−056. −0.134]
Surf Learning—>	Burnout	0.271	[0.197, 0.345]	0.166	[0.067, 0.256]	0.111	[0.065, 0.231]	Partial mediat	[0.065, 0.231]
Surf Learning—>	Regul teach							Non-effect	
Surf Learning—>	Stress Teach							Non-effect	
Surf Learning—>	Stress Learn	0.119	[0.056, 0.123]	0.00	[−0.023, 0.031]	0.119	[0.056, 0.123]	Full mediat	[0.056, 0.123]
Surf Learning—>	Stress Symt	0.106	[0.032, 0.145]	0.00	[−0.011, 0.019]	0.106	[0.032, 0.145]	Full mediat	[0.032, 0.145]
Self–regulation—>	Procrastinat	−0.182	[−0.221, −0.126]	−0.182	[−0.221, −0.126]	0.00	[−0.012, 0.034]	Direct only	[−0.221, −0.126]
Self–regulation—>	Emot coping	−0.008	[−0.001, −0.032]	0.00	[−0.032, 0.134]	−0.008	[−0.001, −0.032]	Partial mediat	[−0.001, −0.032]
Self–regulation—>	Resilience	0.208	[0.325, 0.112]	0.208	[0.325, 0.112]	0.00	[−0.0.04, 0.06]	Direct only	[0.325, 0.112]
Self–regulation—>	Negat. emot							Non-effect	
Self–regulation—>	Acad. confi	0.340	[0.410, 0.225]	0.340	[0.410, 0.225]	0.00	[−0.03, 0.05]	Direct only	[0.410, 0.225
Self–regulation—>	Burnout	−0.084	[−0.012, −0.023]	0.00	[−0.02, 0.08]	−0.084	[−0.012, −0.023]	Full mediat	[−0.012, −0.023]
Self–regulation—>	Regul teach							Non-effect	
Self–regulation—>	Stress Teach							Non-effect	
Self–regulation—>	Stress Learn	−0.021	[−0.032. −0.051	0.00	[−0.04, 0.06]	−0.021	[−0.032. −0.051]	Full mediat	[−0.032. −0.051
Self–regulation—>	Stress Symt	−0.190	[−0.071, 0.224]	−0.142	[−0.243, −0.097]	−0.048	[−0.067, −0.032]	Partial mediat	[−0.067, −0.032]
Procrastination—>	Self–Regul.	−0.129	[−0.023, −0.229]	−0.129	[−0.023, −0.229]	0.00	[−0.012, 0.232]	Direct only	[−0.012, 0.232]
Procrastination—>	Emo. coping	0.217	[0.145, 0.324]	0.170	[0.070, 0.231]	0.047	[−0.02, 0.09]	Partial mediat	[−0.02, 0.09]
Procrastination—>	Resilience	−0.037	[−0.010, −0.32]	0.00	[−0.04, 0.06]	−0.037	[−0.010, −0.32]	Full mediation	[−0.010, −0.32]
Procrastination—>	Negat. Emot	0.284	[0.123, 0.326]	0.237	[0.123, 0.321]	0.047	[0.012, 056]	Partial mediat	[0.012, 056]
Procrastination—>	Acad. confid	−0.044	[−0.021, −0.56]	0.00	[−0.01, 0.07]	−0.044	[−0.021, −0.56]	Full mediat	[−0.021, −0.56]
Procrastination—>	Burnout	0.242	[0.146, 0.356]	0.208	[0.116, 0.267]	0.034	[0.012, 0.045]	Partial mediat	[0.012, 0.045]
Procrastination—>	Regul teach							Non-effect	
Procrastination—>	Stress Teach							Non-effect	
Procrastination—>	Stress Learn	0.142	[0.045, 0.234]	0.00	[−0.04, 0.07]	0.142	[0.045, 0.234]	Full mediat	[0.045, 0.234]
Procrastination—>	Stress Symt	0.277	[0.121, 334]	0.185	[0.113, 0.279]	0.092	[0.04, 23]	Partial mediat	[0.04, 23]
Emotion coping—>	Resilience							Non-effect	
Emotion coping—>	Negat. Emot	160	[0.131, 0.210]	0.160	[0.131, 0.210]	00.	[−0.02, 0.08]	Direct only	[0.131, 0.210]
Emotion coping—>	Acad. confi							Non-effect	
Emotion coping—>	Engagement							Non-effect	
Emotion coping—>	Regul teach							Non-effect	
Emotion coping—>	Stress Teach							Non-effect	
Emotion coping—>	Stress Learn							Non effect	
Emotion coping—>	Stress Symt	0.078	[0.021, 0.123]	0.00	[−0.021, 0.24]	0.078	[0.021, 0.123]	Full mediat	[0.021, 0.123]
Resilience—>	Posit. emot							Non-effect	
Resilience—>	Acad. confi							Non-effect	
Resilience—>	Engagement							Non-effect	
Resilience—>	Regul teach							Non-effect	
Resilience—>	Stress Teach							Non-effect	
Resilience—>	Stress Learn							Non-effect	
Resilience—>	Stress Sympt	−0.262	[−0.123, −0.312]	−0.131	[−0.02, 0.24]	−0.131	[−0.02, −0.24]	Part mediat	[−0.02, −0.24]
Negat. emot—>	Acad. confi							Non-effect	
Negat. emot—>	Burnout	0.026	[−0.03, 0.056]	0.00	[−0.04, 0.07]	0.026	[−0.03, 0.056]	Full mediat	[−0.03, 0.056]
Negat. emot—>	Regul teach							Non-effect	
Negat. emot—>	Stress Teach							Non-effect	
Negat. emot—>	Stress Learn	0.163	[0.114, 0.223]	0.163	[0.114, 0.223]	0.00	[−0.02, 0.04]	Direct only	[0.114, 0.223]
Negat. emot—>	Stress Sympt					0.195			
Regul. Teach—>	Positivity	0.220	[0.12, 0.34]	0.220	[0.12, 0.34]	0.00	[−0.04, 0.10]	Direct only	[0.12, 0.34]
Regul. Teach—>	Deep Learn							Non-effect	
Regul. Teach—>	Self–Regulation	0.107	[0.06, 0.15]	0.107	[0.06, 0.15]	0.00	[−0.04, 0.10]	Direct only	[−0.04, 0.10]
Regul. Teach—>	Procrastinat	−0.111	[−0.07, −0.14]	−0.111	[−0.07, −0.14]	0.00	[−0.07, 0.05]	Direct omly	[−0.07, 0.05]
Regul. Teach—>	Emot.coping	008	[−0.001, 0.010]	0.00	[−0.05, 0.07]	0.008	[−0.001, 0.010]	Partial mediat	[−0.001, 0.010]
Regul. Teach—>	Resilience	0.161	[0.08, 0.23]	0.00	[−0.04, 0.09]	0.161	[−0.02, 0.23]	Partial mediat	[−0.02, 0.23]
Regul. Teach—>	Negat. emot	−0.141	[−0.05, −0.25]	0.00	[−0.05, 0.08]	−0.141	[−0.05, −0.25]	Full mediation	[−0.05, −0.256]
Regul. Teach—>	Acad. confi	0.262	[0.12, 0.34]	0.144	[0.05, 0.25]	0.118	[0.06, 0.23]	Partial mediat	[0.06, 0.23]
Regul. Teach—>	Burnout	−0.229	[−0.12, −0.34]	−0.116	[−0.05,−0.25]	−0.113	[−0.09,−0.23]	Partial mediat	[−0.09,−0.23]
Regul. Teach—>	Stress Teach	−0.100	[−0.05, 0.18]	−0.100	[−0.05, 0.18]	0.00	[−0.04, 08]	Direct only	[−0.05, 0.18]
Regul. Teach—>	Stress Learn	−0.098	[−0.03, −0.21]	0.00	[−0.03, 0.09]	−0.098	[−0.03, −0.21]	Full mediat	[−0.03, −0.21]
Regul. Teach—>	Stress Sympt	−0.113	[−0.06,−0.23]	0.00	[−0.06, 0.10]	−0.113	[−0.06,−0.23]	Full mediat	[−0.06,−0.23]
Stress Teach—>	Positivity							Non-effect	
Stress Teach—>	Deep Learn							Non-effect	
Stress Teach—>	Self–Regulation							Non effect	
Stress Teach—>	Emot. coping	0.397	[0.22, 0.49]	0.338	[0.27, 41]	0.059	[0.02, 0.09]	Partial mediat	[0.02, 0.09]
Stress Teach—>	Resilience							Non-effect	
Stress Teach—>	Negat. emot	0.021	[0.010, 0.035]	0.00	[−0.03, 0.06]	0.021	[0.010, 0.035]	Full mediat	[0.010, 0.035]
Stress Teach—>	Acad. confi							Non-effect	
Stress Teach—>	Burnout	0.107	[0.05, 0.22]	0.107	[0.05, 0.22]	0.00	[−0.04, 0.09]	Direct only	[0.05, 0.22]
Stress Teach—>	Regul teach							Non-effect	
Stress Teach—>	Stress teach							Non-effect	
Stress Teach—>	Stress learn	0.535	[0.612, 0.345]	0.391	[0.421, 0.232]	0.144	[0.08, 0.21]	Part. mediat	[0.08, 0.21]
Stress Teach—>	Stress Sympt	0.261	[0.134, 0.322]	0.00	[−0.21, 0.35]	0.261	[0.134, 0.322]	Full mediat	[0.134, 0.322]
Stress Learn—>	Positivity							Non-effect	
Stress Learn—>	Deep Learn							Non-effect	
Stress Learn—>	Self–Regul							Non-effect	
Stress Learn—>	Emot copin	0.163	[0.102, 0.345]	0.163	[0.102, 0.345]	0.00	[−0.02, 0.34]	Direct only	[−0.02, 0.34]
Stress Learn—>	Resilience							Non-effect	
Stress Learn—>	Negat. emot	0.375	[0.12, 0.45]	0.375	[0.12, 0.45]	0.00	[−0.04., 07]	Direct only	[0.12, 0.45]
Stress Learn—>	Acad. confi							Non-effect	
Stress Learn—>	Engagement							Non-effect	
Stress Learn—>	Regul Teach							Non-effect	
Stress Learn—>	Stress Learn							Non-effect	
Stress Learn—>	Stress Symt	0.487	[0.523, 0.342]	0.487	[0.523, 0.342]	0.00	[−0.04, 0.21]	Direct only	[0.523, 0.342]

*Bootstrapping sample size = 564. Model 4 (Teaching & Learning Factors).*

*Surf Learning, surface approach; Acad. confi, academic behavioral confidence; Emot. Coping or Emotion Coping, emotion-focused coping strategies; Negat. emot, negative achievement emotions; Regul. Teach, regulatory teaching; Stress Teach, stress factors of teaching process; Stress Learn, stress factors of learning process; Stress Sympt, symptoms of stress. CI, confidence interval. Bootstrapping sample size = 430*.

## Discussion

In general, the results support the established hypotheses in various aspects. Regarding Hypothesis 1, certain personality factors (*conscientiousness* and *positivity*) demonstrated their protective function against stress by positively and directly predicting the behaviors that make up the SLPS competency, and negatively and indirectly predicting stress levels. This result concurs with previous research that asserted the factors of *conscientiousness* and *positivity* as *personal factors that protect against stress* (Caprara and Steca, 2005; Caprara et al., 2017; Greene et al., 2020) and as predictors of adequate learning processes (Biggs, 1970b). Previous evidence showed a positive, partially predictive relationship between different factors of the SLPS competency and the variables of deep approach, academic behavioral confidence, problem-focused coping (Leszko et al., 2020), positive emotions, and resilience (de la Fuente et al., 2017a, 2019b).

It is of great interest that behaviors that are inherent in the SLPS competency, according to its model (de la Fuente, 2015a), prove to be associated with and to predict each other, forming clusters of protective (*buffering*) and risk factors. This has revealed the existence of a group of *protective factors* against stress, such as the deep approach, which has a positive linear relationship with academic behavioral confidence, self-regulation, positive achievement emotions, engagement, and resilience, in line with what was reported in previous research (Quoidbach et al., 2010; Artuch-Garde et al., 2017; de la Fuente et al., 2021f). This reflects a clear associative relationship between the metacognitive, meta-behavioral, meta-motivational, emotional, and attitudinal variables of learning, when learning takes place under conditions of university academic stress. These variables have been traditionally separated in their effects and their analyses—just as what is espoused by the SLPS competency model. Finally, the above variables were found to negatively predict, both directly and indirectly, the factors of *stress in learning* and *stress symptoms* in a manner consistent with the previous evidence (Alias et al., 2020).

Hypothesis 2 has also been confirmed since *regulatory* or *effective teaching* directly predicted personal *positivity*, as well as various protective factors of the SLPS competency, such as academic behavioral confidence, engagement, and achievement emotions, as seen in previous research (Baeten et al., 2010). But it is also interesting to note that such teaching also negatively predicted the factor *stress in teaching*, which would lead to a direct negative prediction of *stress in learning*, also established by previous research (de la Fuente and Justicia, 2003). In other words, *regulatory teaching* would indirectly and inversely predict the factor *stress in learning* and, consequently, stress symptoms. This result is consistent with previous research that also established causal factors of stress in the teaching process, and it provides empirical support for the SRL vs. ERL theory (de la Fuente et al., 2020a,c,d,f).

The assumptions of Hypothesis 3 were also empirically supported by our results. The personal factor *neuroticism* was confirmed as a personal risk factor since it minimizes *positivity* (Greene et al., 2020), as well as protective factors of the SLPS competency, such as academic behavioral confidence, self-regulation, and resilience (McDonnell and Semkovska, 2020). *Neuroticism* is also a positive predictor of the risk factors analyzed here, such as surface approach, procrastination, burnout, and negative achievement emotions, as supported by abundant prior evidence (Chen et al., 2020; Yang et al., 2020). In addition, a positive linear relationship between risk factors of the SLPS competency has been demonstrated. Thus, surface approach was shown to be connected through association and prediction to a lack of *academic behavioral confidence* and *self-regulation* (de la Fuente et al., 2013) and to the use of *emotion-focused coping strategies, burnout, negative achievement emotions*, and a lack of *resilience* (de la Fuente et al., 2017b). Therefore, this positive linear connection between meta-cognitive, meta-affective, meta-motivational, emotional, and attitudinal risk factors could be considered a *cluster of risk* for experiencing academic stress, since all of them positively predict *stress in learning* and *stress symptoms*. Despite these results, some authors have defended the potential of stress during university learning (Rudland et al., 2019).

Finally, regarding Hypothesis 4, *regulatory or effective teaching* also appeared as a negative predictor of risk factors for the SLPS competency, such as *procrastination* (Brando-Garrido et al., 2020) and *burnout* in a direct manner but also indirectly through the *stress in teaching* factor, which also predicted *negative achievement emotions* and *stress in learning*. These results are consistent with an integrated vision of teaching and learning processes in the analysis of academic phenomena at a university (Prosser and Trigwell, 1999; Rosário et al., 2013). Based on previous research and the results of the present study, the SLPS competency includes certain *student factors, both presage and process*, that protect against academic stress, while other factors constitute risks for experiencing stress. Moreover, these factors coexist with *presage and process factors of teaching*, which also directly predispose and predict the experience of stress (Biggs and Tang, 2007; Kember et al., 2020; de la Fuente et al., 2021e). This evidence is highly important for university policies of prevention, assessment, and psychoeducational intervention in the emotional health and well-being of students during the COVID-19 period.

Several research *limitations* should be mentioned. On one hand, the sample was limited and may have a selection bias due to the arbitrary selection procedure. On the other hand, the sample was taken at a time prior to the health emergency. The model, then, remains to be validated *in situ*. Unfortunately, the short impact period of the COVID-19 event does not yet allow for a process evaluation. Another methodological limitation when extrapolating consequences from this research study is the fact that the data were not collected over a long period, with a longitudinal design, but were collected under a short, cross-sectional design (the real duration of the university subject). The results from paths that generated a better fit of the selected model cannot be interpreted in causal terms, given that verification, using a longer, longitudinal design, would be required. Another limitation is the molar level of analysis used in this research (de la Fuente et al., 2019b), distant from the biological processes of microanalysis. In the future, the analysis of these relationships should be contextualized within this current moment of a health emergency, and the variable of positive vs. negative *emotional reactivity* should be explored (Becerra et al., 2017), as being particularly important in this competency.

### Implications for Actions During the COVID-19 Emergency

Current events are forcing us to make broad behavioral adjustments in the organization of our personal life, family life, and academic life for the weeks ahead. In order to make these adjustments smoothly, we need to keep in mind different behavioral principles and strategies. For example (de la Fuente, 2020b):

1) Presage factors:
*Students:* It is important to know the characteristics of students to be able to detect which students have protective factors (and so reinforce them) and which are more likely to have personal risk factors (and so be able to intervene). The aim is to keep students from falling into a *vicious circle of risk factors* during the COVID-19 episode due to their low levels of SLPS competency.
*Teachers:* Based on previous evaluations, we need to identify which teaching processes incorporate protective factors against stress (regulatory design) or, instead, involve risk factors in stress (non-regulatory or dysregulatory design). In the former case, these teaching processes should be reinforced and fine-tuned to the new situation, without big changes. In the second case, factors and adjustments that are most dysregulatory toward the learning process must be identified (e.g., drastic changes in content, methodologies, timing, and assessment). These must be avoided or corrected (de la Fuente et al., 2020e).
2) Process factors: Certain intervention strategies are suggested for maximizing the stress-buffering effect of the SLPS competency. Example:

#### Students: Self-Regulation and Self-Regulated Learning

*1) While homebound, stay close to your usual schedule:*
· Circadian rhythms and personal habits go far in helping to maintain a sequence of actions to self-regulate and to not lose motivation.
· Give yourself daily doses of positive emotions and rewarding experiences while sheltering at home. It is very important to keep a positive emotional outlook. *Distress* (diffuse negative emotionality and discouragement) can be triggered by abrupt changes in the daily rhythm of one, or by a sense of uncertainty and loss of behavioral control.
*2) Self-regulate your own behavior during this period*:
· Every day, *plan* objectives, schedules, and actions, being flexible but also systematic.
· Exercise *control* over your own behavior. Force yourself to continue working and also to stop and take leisure time (a substitute for outside activities). Tell yourself that you are doing the right thing. Use different relaxation techniques to decrease any anxiety.
· It is not a good time to take on serious, complex issues in your life situation, because this may cause even greater stress and loss of situational control. If it is truly necessary, make small, gradual adjustments.
·Take advantage to catch up on pending matters, whether personal, family-related or academic tasks. This is a gift of time.
· *Evaluate* your behavior at the end of the day and redefine your objectives (family related, personal, and academic) for the next few days.

#### Teachers: Self-Regulation and External Regulation of Students

*1) In the subjects you teach, maintain a regulatory approach that gives your students a perception of control and continuity:*
**·** Keep your usual hours of contact with the students, using appropriate technology. Direct online classes allow you to continue with the subject and lessen anxiety about the students.
· Make every adjustment you can so that all participants perceive normality and a sense of control. It is best to keep up the regular pace of the subject while making adjustments that the situation requires. It is not a good time to make big, unexpected changes.
· If needed, adjust your assessment activities and system during this period. Make students aware that the new situation means new behavioral challenges, including the chance for them to practice online teamwork from home.
*2) Apply external regulation to help students in their learning process:*
**·** If you have not already done so, this is a good time to convert all learning resources to online formats and encourage students to learn autonomously from home.
· *Plan* regular, general messages and aids for your students so they feel that the teaching-learning process continues with some normality.
· *Offer* personalized online tutoring for students who need it. It is especially important to keep direct contact with the student representative in each class in order to be informed of any possible problems or help that the students are needing.
· Regularly *reevaluate* whether students need adjustments to the material, assignments, etc.
· Pay attention to the emotional state and expectations of your students. Convey calm and assurance with your own behavior. Your students see themselves reflected in you and your demeanor when interacting with them. Become a *mentor* that supports the process, also on an emotional level.
3) Product factors: For students with risk factors or vulnerability to stress, in addition to the steps mentioned above, specific emotional regulation techniques like mindfulness (de la Fuente et al., 2018) or emotional refocusing (Quoidbach et al., 2010) should be worked on, given their effectiveness in the short and long term.

## Conclusion

Empirical models of university academic stress can be useful for: (1) detecting university students who may be at risk during health emergencies like COVID-19; (2) designing psychoeducational learning support systems for students who are experiencing stress in this situation; (3) promoting teaching strategies that protect against academic stress in this context. If we have preventive models of this academic phenomenon, it will be easier to prepare ourselves sooner for emergencies like the one we are currently experiencing. It is very important that certain behavioral repertories be implemented, and so act as psychological vaccines for coping with stress and improving well-being at a university. This is done through psychoeducational programs that improve the competence of students and teachers before the symptoms of academic stress set in (especially in large-scale events like COVID-19) (Coulson, 2019; Young et al., 2020). The services of our University Guidance and Psychology Departments should be an essential, irreplaceable tool for accomplishing this task. The analysis heuristic presented here could be used by Applied Psychology Units at universities for evaluating and intervening in processes of academic stress.

## Data Availability Statement

The raw data supporting the conclusions of this article will be made available by the authors, without undue reservation.

## Ethics Statement

The studies involving human participants were reviewed and approved by The Ethics Committee of the University of Navarra (ref. 2018.170). The patients/participants provided their written informed consent to participate in this study.

## Author Contributions

JdF: conceptual design, data analysis, writing the article, and R&D Project management.

## Conflict of Interest

The author declares that the research was conducted in the absence of any commercial or financial relationships that could be construed as a potential conflict of interest.

## Publisher's Note

All claims expressed in this article are solely those of the authors and do not necessarily represent those of their affiliated organizations, or those of the publisher, the editors and the reviewers. Any product that may be evaluated in this article, or claim that may be made by its manufacturer, is not guaranteed or endorsed by the publisher.
